# Effect of Smartphone-Based Financial Incentives on Peripartum Smoking Among Pregnant Individuals: A Randomized Clinical Trial

**DOI:** 10.1001/jamanetworkopen.2022.11889

**Published:** 2022-05-13

**Authors:** Allison N. Kurti, Tyler D. Nighbor, Katherine Tang, Hypatia A. Bolívar, Carolyn G. Evemy, Joan Skelly, Stephen T. Higgins

**Affiliations:** 1 The University of Vermont, Burlington

## Abstract

This randomized clinical trial assesses the effect of a smartphone-based intervention with financial incentive on peripartum smoking among pregnant individuals.

## Introduction

Cigarette smoking during pregnancy can cause serious adverse pregnancy, birth, and longer-term health outcomes.^1,2^ The most efficacious smoking cessation intervention for peripartum individuals is abstinence-contingent financial incentives (FIs), but there are challenges to scaling this intervention, including reaching individuals in geographically remote areas while retaining treatment efficacy.^3,4^ To address that challenge, this study examined the efficacy of a smartphone-based intervention whereby smoking monitoring and incentive delivery was managed via a mobile app.

## Methods

This randomized clinical trial included 90 pregnant individuals aged 18 years or older who were recruited nationally via social media; obstetrical clinics; and Special Supplemental Nutrition Program for Women, Infants, and Children (WIC) offices between April 2019 and May 2020. The trial protocol appears in Supplement 1. The University of Vermont College of Medicine institutional review board approved this study, and all participants provided written informed consent. The study follows Consolidated Standards of Reporting Trials (CONSORT) reporting guidelines for trial studies (eFigure in Supplement 2).

Participants were randomized to Best Practices (BP) alone or with FIs (BP with FI) (detailed previously).^5^ Briefly, BP included brief counseling and a tobacco quit-line referral. BP with FI included BP plus an FI intervention in which smoking monitoring and incentive delivery were completed via smartphone app (DynamiCare Health Inc). Participants submitted videos of themselves conducting salivary cotinine tests remotely (Alere iScreen [New Line Medical]) and received autogenerated notifications detailing test results and associated earnings. Incentives were delivered from study start to 12 weeks post partum via a debit card using an escalating schedule (maximum earnings, approximately $1620; mean [SD] earnings, $330.52 [$446.18]).^5^

All participants completed 6 assessments during study participation, including a 24-week postpartum assessment after incentives had been discontinued. There were no significant differences between treatment groups in assessment adherence. The primary outcome was 7-day point-prevalence abstinence (self-reported past week abstinence plus a cotinine-negative saliva test). Analyses of smoking-abstinence outcomes included all participants assigned to treatment, except 2 individuals withdrawn following miscarriage. A repeated-measures analysis for categorical data was used to compare treatment conditions on smoking abstinence across assessments based on generalized estimating equations using a logistic link function (SAS PROC GENMOD). Analyses were performed using SAS version 9 (SAS Institute), and a 2-tailed *P* < .05 was considered statistically significant.

## Results

Participant characteristics are provided in the Table. Of 167 eligible individuals who completed an initial study orientation session, 90 were enrolled (48 in the BP group; 42 in the BP with FI) across 33 states.

**Table.** Participant Characteristics Overall and By Treatment Group

Characteristic	Participants, No. (%)		
	Overall (N = 90)	BP with FI (n = 42)	BP alone (n = 48)
Demographic characteristics			
Age, mean (SD), y	31.63 (4.67)	31.25 (4.46)	31.9 (4.87)
Race and ethnicity^a^			
American Indian	0	0	0
Asian or Pacific Islander	0	0	0
Black	16 (18)	9 (23)	7 (15)
Hispanic	4 (5)	1 (2)	3 (6)
Multiple	5 (6)	3 (8)	2 (4)
White	65 (75)	27 (66)	38 (81)
Other^b^	1 (1)	1 (3)	0 (0)
Education, y			
<12	9 (10)	4 (10)	5 (10)
12	42 (47)	20 (49)	22 (46)
>12	38 (43)	17 (41)	21 (44)
Married	26 (29)	11 (27)	15 (31)
Working for pay outside home	46 (52)	22 (54)	24 (50)
Living in a rural county	21 (27)	10 (29)	11 (26)
Participates in WIC	45 (51)	20 (49)	25 (52)
Private insurance	26 (29)	10 (24)	16 (33)
Smoking characteristics			
Age started smoking, mean (SD), y	16.60 (3.70)	15.83 (3.47)	17.25 (3.80)
Cigarettes per day prepregnancy			
<10	9 (10)	6 (15)	3 (6)
>10	80 (90)	35 (85)	45 (94)
Cigarettes per day at intake			
<10	37 (42)	18 (44)	19 (40)
>10	52 (58)	23 (56)	29 (60)
Living with another smoker	56 (63)	28 (68)	28 (58)
No smoking allowed in home	48 (54)	24 (59)	24 (50)
Cigarette type			
Ultra-light or light	16 (18)	7 (17)	9 (19)
Medium	12 (13)	3 (7)	9 (19)
Full flavor	61 (69)	31 (76)	30 (62)
Tried quitting			
Prepregnancy	67 (75)	33 (80)	34 (71)
During pregnancy	57 (64)	26 (63)	31 (65)
Time to first cigarette before pregnancy <5 min after waking	39 (44)	16 (39)	23 (48)
Time to first cigarette at intake <5 min after waking	13 (15)	4 (10)	9 (19)
Utilized tobacco quit line during pregnancy	14 (21)	6 (19)	8 (22)
Pregnancy characteristics			
Gestational age, mean (SD), wk	15.62 (5.63)	13.71 (5.44)	17.29 (5.30)

Abbreviations: BP, best practices; FI, financial incentives; WIC, Special Supplemental Nutrition Program for Women, Infants, and Children.

^a^ Participants classified their race and ethnicity according to categories provided by the investigator.

^b^ Other includes participants who did not belong to any of the investigator-provided categories.

Individuals assigned to BP with FI had nearly 4-fold greater odds of smoking abstinence across antepartum and postpartum assessments compared with individuals receiving BP (χ^2^_1_ = 6.96; adjusted odds ratio, 3.82; 95% CI, 1.63-8.92; *P* = .008) (Figure). Abstinence levels decreased across time (χ^2^_5_ = 16.33; *P* = .006), with odds of abstinence lower at all postpartum assessment vs the initial antepartum assessment; time did not interact significantly with treatment condition (χ^2^_5_ = 4.57; *P* = .47), indicating that abstinence levels in the BP with FI remained greater than those in the BP group through 24 weeks post partum.

**Figure.**
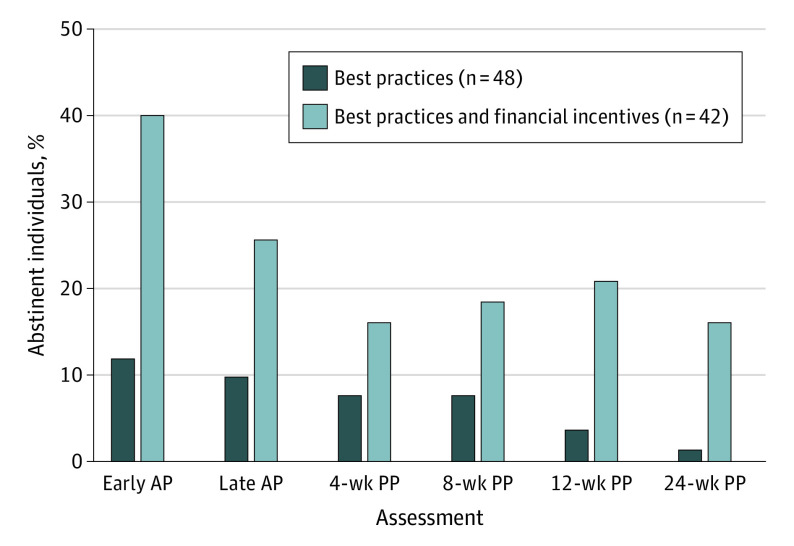
Seven-Day Point-Prevalence Abstinence Rates for Each Treatment Condition Across Antepartum (AP) and Postpartum (PP) Assessments

## Discussion

The nearly 4-fold greater odds of quitting smoking among individuals treated with BP with FI vs BP alone is consistent with meta-analyses identifying FIs as the most effective intervention for peripartum individuals^3,6^ and provides a seminal experimental demonstration that the intervention can be delivered remotely while retaining efficacy comparable with clinic-based outcomes.^4^ This successful treatment of a national, diverse participant sample highlights the capacity for the present innovative treatment delivery platform to expand the reach of this intervention to a broader swath of peripartum individuals, including those with socioeconomic disadvantage, rural residents, Indigenous individuals, and other racial and ethnic minority individuals. Limitations include the potential for internet-based recruitment to generate samples with higher socioeconomic status than recruiting via WIC offices^4^ and reduced precision in estimates of treatment effect size due to underenrollment.

Further research examining cost-effectiveness is an important next step in the development and dissemination of this treatment innovation. The present results underscore the considerable potential of this intervention for increasing peripartum smoking cessation, improving maternal-infant health, and reducing health disparities.

## Notes

Supplement 1.
Trial Protocol

Supplement 2. **eFigure.** Study Flowchart

Supplement 3.
Data Sharing Statement
