# Supplementary material for: Effect of Smartphone-Based Financial Incentives on Peripartum Smoking Among Pregnant Individuals: A Randomized Clinical Trial
Source: JAMA Netw Open. 2022 May 13;5(5):e2211889. doi: 10.1001/jamanetworkopen.2022.11889 (PMC9107025; doi:10.1001/jamanetworkopen.2022.11889)
Supplement: Supplement 1. — Trial Protocol [file jamanetwopen-e2211889-s001.pdf]

The University of Vermont Committees on Human Research

For Committee Use Only
PROTOCOL NUMBER

Human Subjects Research Protocol

The Common Human Subjects Protocol Cover Form **must** be completed and **attached** to the front of this form. This Protocol form should be completed for any human subjects research proposal that does not have a specific "protocol," such as a grant application. This form must be submitted along with a copy of the complete grant proposal and all the information in this form **must** be consistent with that proposal. This protocol form, once IRB approved, will be the working protocol for that research. **When completing this document, do not refer to page numbers within your grant**. If revisions are necessary during the course of the research, amendments should refer to this protocol form, not the grant proposal. Enter responses for all sections. Check N/A if the section does not apply.

PROTOCOL SUMMARY

Project Title:	Protocol Version Date:
Smartphone-based Financial Incentives to Promote Smoking Cessation Among Pregnant Women	4/2/2020

Principal Investigator: Stephen T. Higgins, Ph.D.

Grant Sponsor: NIGMS

Grant Number: 4P20GM103644-04
(For grants routed through UVM, indicate the OSP Proposal ID # located at the top of the OSP Routing Form)

Lay Language Summary: *(Please use non-technical language that would be understood by nonscientific IRB members to summarize the proposed research project. The information must include: (1) a brief statement of the problem and related theory supporting the intent of the study, and (2) a brief but specific description of the procedure(s) involving the human subjects. Please do not exceed one single-spaced 8 ½ X 11" page.)*

Cigarette smoking during pregnancy increases risk for catastrophic pregnancy complications, growth retardation, other adverse fetal and infant health problems, and later-in-life chronic conditions among exposed offspring. The most effective intervention for reducing smoking during pregnancy is financial incentives whereby participants earn incentives (e.g., gift cards, cash) contingent on objective evidence of smoking abstinence. However, financial incentives-based interventions are typically delivered in relatively intense protocols requiring frequent clinic visits, which limits the geographical range over which services can be delivered and potentially denies treatment to those residing in remote or otherwise difficult to reach settings.

The present study will examine the feasibility, efficacy, and cost-effectiveness of a smartphone-based financial incentives intervention whereby smoking monitoring and delivery of incentives are completed remotely using a mobile app (to be designed by DynamiCare Health, Inc.). Pregnant women will be recruited via obstetrical clinics, WIC offices, and ads in print and online media outlets in all Vermont counties, and in obstetrical clinics and WIC offices outside Vermont. Eligible participants who complete the informed consent process will be randomized to one of two conditions: an incentives condition wherein women will receive financial incentives contingent on the remote submission of breath and saliva specimens indicating abstinence from recent smoking (described below), or a best practices control condition in which women will receive usual care for smoking cessation that is provided at their obstetrical clinics, as well as three brief educational sessions and referral to the Vermont (or other state) pregnancy-specific quit line by our research staff. This is the same control condition that we our using in our current ongoing cost-effectiveness trial (CHRMS 13-143).

For inclusion in the study, women must meet the following criteria: (a) $\geq$ 18 years of age, (b) report being smokers at the time they learned of the current pregnancy, (c) report smoking in the 7 days prior to completing their phone eligibility screening, (c) $\leq$ 25 weeks pregnant, (d) speak English, (e) own a smartphone (Android or iOS; 81.8% of pregnant women in wave 1 [2013-2014] of the Population Assessment of Tobacco and Health [PATH] reported owning a smartphone). Exclusion criteria include: (a) current or prior mental or medical condition that may interfere with study participation (assessed via self-report during phone eligibility screening), (b) smoke marijuana more than once each week and not willing to quit (marijuana smoking can inflate breath CO), (c) exposed to unavoidable occupational sources of CO (e.g., car mechanic), and (d) self-report currently being maintained on

CHRMS (Medical) #17-0604 Approved: 4/3/2020

opioid maintenance therapy (e.g., methadone, buprenorphine).

Participants randomized to the incentives condition will select a quit date (either the first or second Monday following their enrollment), and will submit videos of themselves blowing into a breath CO monitor twice daily during week 1. They will receive incentives for every sample where expired breath CO is ≤ 6 ppm. Beginning in week 2 and extending through week 6, participants will submit videos twice per week (Monday/Thursday) for which they will receive incentives for providing videos of themselves completing saliva cotinine tests indicating smoking abstinence. From week 7 until delivery, participants will submit videos once per week and will continue to receive incentives for saliva cotinine tests indicating no smoking. During the postpartum period, women will submit videos twice weekly for the first 4 weeks and once weekly from weeks 5-12. The first negative breath CO or salivary cotinine sample is worth $6.25 and each consecutive negative sample will increase the value of the incentive by $1.00 up to a maximum of $33.25 per sample. Missed samples or samples that indicate smoking will be worth $0 and will reset the incentive at its starting value. However two consecutive negative samples following the slip will restore the incentive to its value before the slip. The maximum earnings in this condition is $1,620, which is the same as our prior studies adjusting for inflation. A proposed voucher schedule is attached.

Women in both conditions will complete seven formal assessments of their smoking status during their participation (intake, early pregnancy or 1 month after enrollment, late pregnancy or ≥ 28 weeks gestation, and at 4-, 8-, 12-, and 24-weeks postpartum) along with a treatment acceptability questionnaire and semi-structured interview on barriers and facilitators of treatment engagement. Women will receive $50 for completing each of these ($400 total)

We conducted a power analysis to estimate the number of participants required to detect treatment effects assuming late-pregnancy abstinence rates of approximately 40% vs. < 10% (incentives vs. best practices, respectively), and 24-week postpartum abstinence rates of approximately 20% vs. < 5%. These abstinence rates are based on data from our current trial and previous trial data indicating a 20% decrease in abstinence rates from late pregnancy to 24-weeks postpartum. The proposed sample size of 76 per treatment condition will result in at least 80% power to detect a difference between the two treatment conditions in abstinence rates of 40% vs. 10 % at late-pregnancy or 20% vs. 5% at 24-weeks postpartum assessments using a chi-square test and significance level of 0.05.

Pregnant women will be recruited via obstetrical clinics, WIC offices, and ads in print and online media outlets in all Vermont counties, and in obstetrical clinics and WIC offices outside Vermont. Recruitment materials that we propose distributing at these sites are attached. We have also attached the battery of questionnaires that we propose administering at formal assessments, as well as the Research Information Sheet that we will read to participants over the phone to obtain verbal consent to participate in the study, as well as the full consent form that we will mail to them after obtaining their verbal consent to participate.

PURPOSE AND OBJECTIVES

Purpose: *The importance of the research and the potential knowledge to be gained should be explained in detail. Give background information.*

Smoking during pregnancy is the leading preventable cause of poor pregnancy outcomes in the U.S. and other developed countries, increasing risk for pregnancy complications, preterm birth, stillbirth, infant death, impaired lung development, childhood illness and developmental/behavioral problems, and lifelong increased risk for cardiovascular disease, obesity, and metabolic syndrome.[1-8] Prevalence of smoking during pregnancy among national samples of U.S. pregnant women has remained stagnant at approximately 13% over the past decade.[9-10] Because risk for maternal smoking during pregnancy is disproportionately high among economically disadvantaged women, it contributes substantially to the problem of health disparities.[11-12]

Existing treatments for smoking cessation among pregnant smokers produce very low quit rates (< 15%) with the exception of financial incentives. Meta-analyses indicate that incentive-based treatments produce the largest effect sizes of any psychosocial or pharmacological intervention for promoting smoking cessation during pregnancy.[13-14] Additionally, two randomized, controlled trials conducted by our research group demonstrated that incentives significantly increase sonographically estimated fetal growth, increasing abdominal circumference (cm/week), femur length (cm/week), and overall estimated fetal weight compared to control groups that received non-contingent incentives.[15] Retrospective analyses of birth outcome data aggregated across three previous trials from our group also indicated that incentives significantly increased mean birth weight, decreased the percent of low birth weight deliveries (< 2500 g), increased the percent of women breastfeeding through 12-weeks postpartum, and decreased depressive symptoms from birth through 12-weeks postpartum among depression-prone women.[16-18] Although this evidence-based treatment has demonstrable capacity to reduce smoking and improve birth outcomes, the scalability of this approach is constrained by the frequent clinic visits necessary for

CHRMS (Medical) #17-0604 Approved: 4/3/2020

biochemical verification of smoking status, which limits access to those in the immediate vicinity of clinics that can provide such care. ***Capitalizing on technological advancements may surmount such access barriers, with the potential to extend the reach of financial incentives to disadvantaged pregnant smokers nationwide.***

The overarching aim of the current study is to develop an innovative, efficacious, remotely delivered financial incentives intervention to reduce cigarette smoking during pregnancy. To enable flexible use of the intervention in diverse locations, we will use a mobile-phone-based platform for delivering the incentives intervention. Recent data indicate that over 80% of pregnant women drawn from a U.S. nationally representative sample own a smartphone.[19] The platform will involve a Smartphone "app" which uses video capture to verify smoking status via a breath carbon monoxide (CO) monitor along with saliva cotinine test kits, and ensures treatment fidelity by providing automated, immediate feedback, incentive calculations, and incentive delivery for abstinence. Comparable technology-based treatment delivery platforms have been used to successfully promote smoking abstinence among the general population of U.S. smokers and among other vulnerable populations including rural heavy smokers.[20]

The proposed study addresses the following specific aims:

Aim 1: Examine the feasibility, efficacy, and cost-effectiveness of a mobile-phone-based incentives intervention for promoting smoking cessation among economically disadvantaged (i.e., Medicaid insured) pregnant women. We will accomplish this aim by randomly assigning 152 pregnant women who are still smoking at their first prenatal care visit to receive incentives contingent on biochemically verified smoking abstinence or best practices for promoting smoking cessation. Incentives will be in place from the start of prenatal care through three months postpartum. Primary outcomes will be point prevalence smoking abstinence, continuous abstinence during and following pregnancy, and birth outcomes (mean birth weight, gestational age, NICU admissions). We hypothesize that there will be higher abstinence rates, longer durations of abstinence, and improved birth outcomes in the incentives versus the best practices condition.

Aim 2: Evaluate acceptability of the treatment, including barriers to and facilitators of treatment engagement. We will accomplish this aim by assessing treatment enrollment and retention and conducting semi-structured interviews with participants upon treatment completion. During these interviews, participants will be queried about their perceived utility of the incentives for promoting health-related behavior change, and other barriers and facilitators of treatment engagement and success (e.g., social support networks).[21-22] Qualitative research methods will be used to discern general themes.

Overall, this project has the potential to address disparities in access to efficacious, evidence-based smoking cessation treatments among economically disadvantaged pregnant women. If the present mobile-phone-based incentives intervention is acceptable and efficacious, this study will provide strong preliminary data for a future R01 proposal to facilitate more widespread dissemination of this innovative treatment model.

***References**. Include references to prior human or animal research and references that are relevant to the design and conduct of the study.*

1. Barker, D.J. (2004). The developmental origins of adult disease. J Am Coll Nutr, 23, 588S-595S.

2. Cohen, G., Jeffery, H., Lagercrantz, H., Katz-Salamon, M. (2010). Long-term reprogramming of cardiovascular function in infants of active smokers. Hypertension, 55, 722-728.

3. Dietz, P.M., England, L.J., Shapiro-Mendoza, C.K., Tong, V.T., Farr, S.L., & Callaghan, W.M. (2010). Infant morbidity and mortality attributable to prenatal smoking in the U.S. Am J Prev Med, 39, 45-52.

4. Rogers, J.M. (2009). Tobacco and pregnancy. Reproductive Toxicology, 28, 152-160.

5. Thompson BL, Levitt P, Stanwood, GD. Prenatal exposure to drugs: effects on brain development and implications for policy and education. Nat Rev Neurosci 2009;10:303-312.

6. Baba S, Wikstom AK, Stephansson O, Cnattingius S. Influence of smoking and snuff cessation on preterm birth. Eur J Epidemiol 2012;27:297-304.

7. U.S. Department of Health and Human Services. The health consequences of smoking—50 years of progress: A report of the Surgeon General. Atlanta, GA: U.S. Department of Health and Human Services, Centers for Disease Control and Prevention, National Center for Chronic Disease Prevention and Health Promotion, Office on Smoking and Health; 2014.

8. Leslie FM. Multigenerational epigenetic effects of nicotine on lung function. BMC Med 2013;11:27.

9. Alshaarawy, O., & Anthony, J. C. (2015). Month-wise estimates of tobacco smoking during pregnancy for the United

CHRMS (Medical) #17-0604 Approved: 4/3/2020
States, 2002–2009. *Maternal and Child Health Journal*, 19(5), 1010-1015.

10. Kurti, A.N., Redner, R., Lopez, A.A., Keith, D.R., Stanton, C.A., Gaalema, D.E., &…Higgins, S.T. (in press). Tobacco and Nicotine Delivery Product Use in a National Sample of Pregnant Women. *Preventive Medicine.*

11. Higgins, S.T., Chilcoat, H.D. (2006). Women and smoking: An interdisciplinary examination of socioeconomic influences. Drug Alcohol Depend, 104 (Suppl. 1), S1-5.

12. Kandel, D.B., Griesler, P.C., & Schaffran, C. (2009). Educational attainment and smoking among women: Risk factors and consequences for offspring. Drug Alcohol Depend, 104 (Suppl. 1), S24-33.

13. Lumley, J., Chamberlain, C., Dowswell, T., Oliver, S., Oakley, L., Watson, L. (2009). Interventions for promoting smoking cessation during pregnancy. Cochrane Database Syst Rev. Jul 8;(3):CDC001055.

14. Chamberlain, C., O'Mara-Eves, A., Porter, J., Coleman, T., Perlen, S.M., & McKenzie, J.E. (2017). Psychosocial interventions for supporting women to stop smoking in pregnancy (review). Cochrane Database Syst Rev., CDC001055.

15. Higgins, S.T., Washio, Y., Lopez, A.A., Heil, S.H., Solomon, L.J., Lynch, M.E., Hanson, J.D., Higgins, T.M., Skelly, J.M., Redner, R., & Bernstein, I.M. (2014). Examining two different schedules of financial incentives for smoking cessation among pregnant women. Prev Med, 68, 51-57.

16. Higgins, S.T., Bernstein, I.M., Washio, Y., Heil, S.H., Badger, G.J., Skelly, J.M., Higgins, T.M., & Solomon, L.J. (2010a). Effects of smoking cessation with voucher-based contingency management on birth outcomes. Addiction, 105, 2023-2030.

17. Higgins, T.M., Higgins, S.T., Heil, S.H., Badger, G.J., Skelly, J.M., Bernstein, I.M., Solomon, L.J., Washio, Y., & Preston, A.M. (2010b). Effects of cigarette smoking cessation on breastfeeding duration. Nic Tob Res, 12, 483-488.

18. Lopez, A.A., Skelly, J.M., & Higgins, S.T. (2015). Financial incentives for smoking cessation among depression-prone pregnant and newly postpartum women: Effects on smoking abstinence and depression ratings. Nicotine Tob Res, 17, 455-462.

19. Kurti, A.N. Unpublished raw data. Population Assessment of Tobacco & Health (PATH) (2013-2014).

20. Stoops, W.W., Dallery, J., Fields, N.M., Nuzzo, P.A., Schoenberg, N.E., Martin, C.A., Casey, B., & Wang, C.J. (2009). An internet-based abstinence reinforcement smoking cessation intervention in rural smokers. Drug Alcohol Depend, 105, 56-62.

21. Bonevski, B., Bryant, J., Lynagh, M., & Paul, C. (2012). Money as motivation to quit: A survey of a non-random Australian sample of socially disadvantaged smokers' views of the acceptability of cash incentives. Prev Med, 55, 122-126.

22. Flemming, K., McCaughan, D., Angus, K., & Graham, H. (2015). Qualitative systematic review: Barriers and facilitators to smoking cessation experienced by women in pregnancy and following childbirth. J Adv Nurs, 71, 1210-1226.

23. LeClere, F.L. & Wilson, J.B. (1990). Smoking behavior of recent mothers, 18-44 years of age, before and after pregnancy: United States, 1990. Adv Data, 288, 1-11.

24. Higgins, S.T., Heil, S.H., Badger, G.J., Skelly, J.M., Solomon, L.J., & Bernstein, I.M. (2009). Educational disadvantage and cigarette smoking during pregnancy. Drug Alcohol Depend, 104, 100-105.

25. Tong, V.T., Dietz, P.M., Morrow, B., D'Angelo, D.V., Farr, S.L., Rockhill, K.M., et al. (2013). Trends in smoking before, during, and after pregnancy-pregnancy risk assessment monitoring system, United States, 40 sites, 2000-2010. MMWR Surveill. Summ., 62, 1-19.

26. U.S. Department of Health and Human Services: The health consequences of smoking: 50 years of progress. A report of the Surgeon General.: U.S. Department of Health and Human Services, Centers for Disease Control and Prevention, National Center for Chronic Disease Prevention and Health Promotion. Office on Smoking and Health, 2014.

27. Cnattingius, S. (2004). The epidemiology of smoking during pregnancy: Smoking prevalence, maternal characteristics, and pregnancy outcomes. Nicotine Tob Res, 6, 125-140.

28. Cohen, R.T., Raby, B.A., Van Steen, K., Fuhlbrigge, A.L., Celedon, J.C., Rosner, B.A., Strunk, R.C., Zeiger, R.S., & Weiss, S.T. (2010). In utero smoke exposure and impaired response to inhaled corticosteroids in children with asthma. J Allergy Clin Immunol, 126, 491-497.

29. Monteiro, P.O., & Victora, C.G. (2005). Rapid growth in infancy and childhood and obesity in later life—a systematic review. Obes Rev, 6, 143-154.

CHRMS (Medical) #17-0604 Approved: 4/3/2020

30. Dennison, B.A., Edmunds, L.S., Stratton, H.H., & Pruzek, R.M. (2006). Rapid infant weight gain predicts childhood overweight. Obes, 14, 491-499.

31. Chrestani, M.A., Santos, I.S., Horta, B.L., Dumith, S.C., Souza, M.A., & Dode, O. (2013). Associated factors for accelerated growth in childhood: A systematic review. J Matern Child Health, 17, 512-519.

32. Buka, S.L., Shenassa, E.D., & Niaura, R. (2003). Elevated risk of tobacco dependence among offspring of mothers who smoked during pregnancy: A 30-year prospective study. Am J Psychiatry, 160, 1978-1984.

33. Atluri, P., Fleck, M.W., Shen, Q., Mah, S.J., Stadfelt, D., Barnes, W., et al. (2001). Functional nicotine acetylcholine receptor expression in stem and progenitor cells of the early embryonic mouse cerebral cotex. Dev Biol, 240, 143-156.

34. Dwyer, J.B., Broide, R.S., & Leslie, F.M. (2008). Nicotine and brain development. Birth Defects Res. C: Embryo Today, 84, 30-34.

35. England, L.J., Aagaard, K., Bloch, M., Conway, K., Cosgrove, K., Grana, R., et al. (2017). Developmental toxicity of nicotine: A transdisciplinary synthesis and implications for emerging tobacco products. Neurosci Biobehav Rev, 72, 176-189.

36. Mohlman, M.K., Levy, D.T. (2016). Disparities in maternal and child health outcomes attributable to prenatal tobacco use. Matern Child Health J, 20, 701-709.

37. Sexton, M. & Hebel, J.R. (1984). A clinical trial of change in maternal smoking and its effect on birth weight. JAMA, 251, 911-915.

38. Bickel, W.K., Johnson, M.W., Koffanrus, M.N., MacKillop, J., Murphy, J.G. (2014). The behavioral economics of substance use disorders: Reinforcement pathologies and their repair. Annu Rev Clin Psychol, 10, 641-677.

39. Bickel, W.K., Odum, A.L., & Madden, G.J. (1999). Impulsivity and cigarette smoking: Delay discounting in current, never, and ex-smokers. Psychopharmacol, 146, 447-454.

40. Bickel, W.K., Jarmolowicz, D.P., Mueller, E.T., Koffanrus, M.N., & Gatchalian, K.M. (2012). Excessive discounting of delayed reinforcers as a trans-disease process contributing to addiction and other disease-related vulnerabilities: Emerging evidence. Pharmacol Ther, 134, 287-297.

41. Jaroni, J.L., Wright, S.M., Lerman, C., & Epstein, L.H. (2004). Relationship between education and delay discounting in smokers. Addict Behav, 29, 1171-1175.

42. Higgins, S.T., Delaney, D.D., Budney, A.J., Bickel, W.K., Hughes, J.R., Foerg, F., & Fenwick, J.W. (1991). A behavioral approach to achieving initial cocaine abstinence. Am J Psychiatry, 148, 1218-1224.

43. Lussier, J.P., Heil, S.H., Mongeon, J.A., Badger, G.J., & Higgins, S.T. (2006). A meta-analysis of voucher-based reinforcement therapy for substance use disorders. Addiction, 101, 192-203.

44. Davis, D.R., Kurti, A.N., Skelly, J.M., Redner, R., White, T.J., & Higgins, S.T. (2016). A review of the literature on contingency management in the treatment of substance use disorders, 2009-2014. Prev Med, 92, 36-46.

45. Higgins, S.T., Heil, S.H., Solomon, L.J., Bernstein, I.M., Lussier, J.P., Abel, R.L., Lynch, M.E., & Badger, G.J. (2004). A pilot study on voucher-based incentives to promote abstinence from cigarette smoking during pregnancy and postpartum. Nicotine Tob Res, 6, 1015-1020.

46. Heil, S.H., Higgins, S.T., Bernstein, I.M., Solomon, L.J., Rogers, R.E., Thomas, C.S., Badger, G.J., & Lynch, M.E. (2008). Effects of voucher-based incentives on abstinence from cigarette smoking and fetal growth among pregnant women. Addiction, 103, 1009-18.

47. Higgins, S.T. Unpublished trial results reported in Higgins, S.T., Washio, Y., Heil, S.H., et al. Financial incentives for smoking cessation among pregnant and newly postpartum women. Prev Med, 2012, S33-40.

48. Higgins, S.T., Washio, Y., Heil, S.H., et al. (2012). Financial incentives for smoking cessation among pregnant and newly postpartum women. Prev Med, 55, S33-40.

49. Ondersma, S.J., Svikis, D.S., Lam, P.K., Connors-Burge, V.S., Ledgerwood, D.M., & Hopper, J.A. (2012). A randomized trial of computer-delivered brief intervention and low-intensity contingency management for smoking during pregnancy. Nicotine Tob Res, 14, 351-360.

50. International Telecommunications Union (2016). ICT Facts and Figures 2016. http://www.itu.int/en/ITU-D/Statistics/Pages/facts/default.aspx

51. Pew Research Center (2016). Mobile Facts and Figures. http://www.pewinternet.org/fact-sheet/mobile/

52. Dallery, J., Glenn, I.M., & Raiff, B.R. (2007). An internet-based abstinence reinforcement treatment for cigarette smoking. Drug Alcohol Depend, 86, 230-238.

53. Dallery, J., Raiff, B.R., & Grabinski, M.J. (2013). Internet-based contingency management to promote smoking cessation: A randomized controlled study. J Appl Behav Anal, 46, 750-764.

54. Dallery, J., Raiff, B.R., Kim, S.J., Marsch, L.A., Stitzer, M., & Grabinski, M.J. (2016). Nationwide access to an internet-based contingency management intervention to promote smoking cessation: A randomized controlled trial. Addiction, doi: 10.1111/add.13715. [Epub ahead of print]

55. Kurti, A.N., Davis, D.R., Skelly, J.M., Redner, R., & Higgins, S.T. (2016). Comparison of nicotine dependence indicators in predicting quitting among pregnant smokers. Exp Clin Psychopharmacol, 24, 12-17.

56. Methodology Committee of the Patient-Centered Outcomes Research Institute (PCORI) (2012). Methodological standards and patient-centeredness in comparative effectiveness research: The PCORI perspective. JAMA, 307, 1636-1640.

57. Whittaker, R., Merry, S., Dorey, E., & Maddison, R. (2012). A development and evaluation process for mHealth interventions: Examples from New Zealand. J Health Commun, 17, 11-21.

58. Naughton, F., Jamison, J., & Sutton, S. (2013). Attitudes towards SMS text message smoking cessation support: A qualitative study of pregnant smokers. Health Educ Res, 28, 911-922.

59. Sloan, M., Hopewell, S., Coleman, T., Cooper, S., & Naughton, F. (2017). Smoking cessation support by text message during pregnancy: A qualitative study of views and experiences of the MiQuit intervention. Nicotine Tob Res, 572-577.

60. Rodger, D., Skuse, A., Wilmore, M., Humphreys, S., Dalton, J., Flabouris, M., & Clifton, V.L. (2013). Pregnant women's use of information and communications technologies to access pregnancy-related health information in South Australia. Aust J Prim Health, 19, 308-312.

61. Kurti, A.N., Logan, H., Manini, T., & Dallery, J. (2015). Physical activity behavior, barriers to physical activity, and opinions about a smartphone-based physical activity intervention among rural residents. Telemedicine and eHealth, 21, 1-8.

62. Kurti, A.N., & Dallery, J. (2014). Integrating technological advancements in health-based behavior interventions: Unprecedented opportunities for behavior analysts. Mex J Behav Anal, 40, 106-126.

63. Dallery, J., Kurti, A.N., & Erb, J.P. (2014) A new frontier: Integrating behavioral and digital technology to promote health behavior. The Behavior Analyst, 38, 19-49.

64. Kurti, A.N., & Dallery, J. (2013) Internet-based contingency management increases walking in sedentary adults. J Appl Behav Anal, 46, 568-581.

65. Dallery, J., Kurti, A.N., & Martner, S. (2014). Technological approaches to assess and treat cigarette smoking. In L. Marsch, S. Lord, & J. Dallery (Eds), *Transforming Behavioral Health Care with Technology: The State of the Science.* Oxford University Press.

66. Kurti, A.N., Keith, D.R., Noble, A., Priest, J.S., & Higgins, S.T. (2016). Co-occurring risk factors for illicit drug abuse and dependence in a U.S. nationally representative sample. Prev Med, 92, 118-125.

67. Kurti, A.N., Klemperer, E., Zvorsky, I., Redner, R., Priest, J., & Higgins, S.T. (2016). Some context for understanding the place of the GED in the relationship between educational attainment and smoking prevalence. Prev Med, 92, 141-147.

68. Higgins, S.T., Kurti, A.N., Redner, R., White, T.J., Keith, D.R., Gaalema, D.E., Roberts, M.E., Doogan, N.J., & Stanton, C.A. (2015). Co-occurring risk factors for current cigarette smoking in a U.S. nationally representative sample. Prev Med, 92, 110-117.

69. Kurti, A.N., Davis, D., Skelly, J.M., Redner, R. & Higgins, S.T (2016). Comparison of nicotine dependence indicators in predicting quitting among pregnant smokers. Exp Clin Psychopharm, 24, 12-17.

70. Kurti, A.N., & Dallery, J. (2014). A laboratory-based evaluation of exercise plus contingency management for reducing cigarette smoking. Drug Alc Depend, 144, 201-209.

71. Kurti, A.N., & Dallery, J. (2014). Effects of exercise on craving and cigarette smoking in the human laboratory. Addict Behav, 39, 1131-1137.

CHRMS (Medical) #17-0604 Approved: 4/3/2020

72. Kurti, A.N., Davis, D., Redner, R., Jarvis, B., Zvorsky, I., Atwood, G.A., &…Higgins, S.T. (2016). Incorporating technology into incentive-based interventions to promote health-related behavior change: A systematic literature review, 2004-2015. Trans Issues Psychol Sci, 2, 128-152.

73. Higgins, S.T., Reed, D.R., Redner, R., Skelly, J.M., Zvorsky, I.Z., & Kurti, A.N. (2016). Simulating demand for cigarettes among pregnant women: A low-risk method for studying vulnerable populations. J Exp Anal Behav, doi: 10.1002/jeab.232

74. Villanti, A., Boulay, M., & Juon, H.S. (2011). Peer, parent, and media influences on adolescent smoking by developmental stage. Addict Behav, 36, 133-136.

75. Villanti, A.C., Richardson, A., Vallone, D.M., & Rath, J.M. (2013). Flavored tobacco product use among U.S. young adults. Am J Prev Med, 44, 388-391.

76. Villanti, A.C., Cantrell, J., Pearson, J.L., Vallone, D.M., & Rath, J.M. (2014). Perceptions and perceived impact of graphic cigarette health warning labels on smoking behavior among U.S. young adults. Nicotine Tob Res, 16, 469-477.

77. Villanti, A.C., Pearson, J.L., Cantrell, J., Vallone, D.M., & Rath, J.M. (2015). Patterns of combustible tobacco use in U.S. young adults and potential response to graphic cigarette health warning labels. Addict Behav, 42, 119-125.

78. Villanti, A.C., Giovino, G.A., Barker, D.C., Mowery, P.D., et al. (2012). Menthol brand switching among adolescents and young adults in the National Youth Smoking Cessation Survey. Am J Public Health, 102, 1310-1312.

79. Rath, J.M., Villanti, A.C., Williams, V.F., et al. (2015). Patterns of longitudinal transitions in menthol use among U.S. young adult smokers. Nicotine Tob Res, 17, 839-846.

80. Villanti, A.C., Rath, J.M., Williams, V.F., et al. (2016). Impact of exposure to electronic cigarette advertising on susceptibility and trial of electronic cigarettes and cigarettes in U.S. young adults: A randomized controlled trial. Nicotine Tob Res, 18, 1331-1339.

81. Heil, S.H., Herrmann, E.S., Badger, G.J., Solomon, L.J., Bernstein, I.M., & Higgins, S.T. (2014). Examining of the timing of changes in cigarette smoking upon learning of pregnancy. Prev Med, 68, 58-61.

82. Vurbic, D., Higgins, S.T., McDonough, S.R., Skelly, J.M., & Bernstein, I.M. (2014). Maternal body mass index moderates the influence of smoking cessation on breastfeeding. Nicotine Tob Res, 16, 527-535.

83. Gaalema, D.E., Higgins, S.T., Pepin, C.S., Heil, S.H., & Bernstein, I.M. (2013). Illicit drug use among pregnant women enrolled in treatment for cigarette smoking cessation. Nicotine Tob Res, 15, 987-991.

84. Mullen, P.D., Carbonari, J.P., Tabak, E.R., & Glenday, M.C. (1991). Improving disclosure of smoking by pregnant women. Am J Obstet Gynecol, 165, 409-413.

85. Javors, M.A., Hatch, J.P., & Lamb, R.J. (2005). Cut-off levels for breath carbon monoxide as a marker for cigarette smoking. Addiction, 100, 159-167.

86. Higgins, S.T., Heil, S.H., Badger, G.J., Mongeon, J.A., Solomon, L.J., McHale, L., & Bernstein, I.M. (2007). Biochemical verification smoking status in pregnant and recently postpartum women. Exp Clin Psychopharmacol, 15, 58-66.

87. Tappin, D., Bauld, L., Purves, D., Boyd, K., Sinclair, L., MacAskill, S.,&…Coleman, T. (2015). Financial incentives for smoking cessation in pregnancy: Randomised controlled trial. BMJ, 350, h134.

88. Fiore, M.C., Bailey, W.C., Cohen, S.J., et al. (2008). Treating Tobacco Use and Dependence. U.S. Department of health and Human Services, Public Health Service. Rockville, MD. (Original manual published in 2000).

89. Roll, J.M., & Higgins, S.T. (2000). A within-subject comparison of three different schedules of reinforcement of drug abstinence using cigarette smoking as an exemplar. Drug Alcohol Depend, 58, 103-109.

90. Kazdin, A.E., & Polster, R. (1973). Intermittent token reinforcement and response maintenance in extinction. Behav Ther, 4, 386-391.

91. Mackintosh, N.J. (1974). The psychology of animal learning. London: Academic Press, 1974.

92. Fagerstrom, K.O., & Schneider, N.G. (1989). Measuring nicotine dependence: A review of the Fagerstrom Tolerance Questionnaire. J Behav Med, 12, 159-182.

93. Brown, R.A., Burgess, E.S., Sales, S.D., Whiteley, J.A., Evans, D.M., & Miller, I.W. (1998). Reliability and validity of a smoking timeline follow-back interview. Psychol Addict Behav, 12, 101-112.

CHRMS (Medical) #17-0604 Approved: 4/3/2020

94. Derogatis, L.R. (1993). *Brief Symptom Inventory: Administration, scoring, and procedures manual*. National Computer Systems (NCS).

95. Beck, A.T., & Beck, R.W. (1972). Screening depressed patients in family practice: A rapid technic. Postgrad Med, 52, 81-85.

96. Johnson, M.W., & Bickel, W.K. (2002). Within-subject comparison of real and hypothetical money rewards in delay discounting. J Exp Anal Behav, 77, 129-146.

97. Jacobs, E.A., & Bickel, W.K. (1999). Modeling consumption in the clinic using simulation procedures: Demand for heroin and cigarettes in opioid-dependent outpatients. Exp Clin Psychopharmacol, 7, 412-426.

98. French, M.T., Roebuck, M.C., & McLellan, A.T. (2004). Cost estimation when time and resources are limited: The Brief DATCAP. J Subst Abuse Treat, 27, 187-193.

99. French, M.T., Fang, H., & Fretz, R. (2010). Economic evaluation of a prerelease substance abuse treatment program for repeat criminal offenders. J Subst Abuse Treat, 38, 31-41.

100. Armitage, P. (1983). Trials and errors: The emergence of clinical statistics. J Roy Stat Soci, 146, 321-334.

101. Boyd, K.A., Briggs, A.H., Bauld, L., Sinclair, L., & Tappin, D. (2015). Are financial incentives cost-effective to support smoking cessation during pregnancy? Addiction, 111, 360-370.

102. Drummond, M., Manca, A., & Sculpher, M. (2005). Increasing the generalizability of economic evaluations: Recommendations for the design, analysis, and reporting of studies. Int J Technol Assess Health Care, 21, 165-171.

103. Goszczynska, E., Knol-Michalowska, K., & Petrykowska, A. (2016). How do pregnant women justify smoking? A qualitative study with implications for nurses' and midwives' anti-tobacco interventions. J Adv Nurs, 72, 1567-1578.

104. Braun, V., & Clarke, V. (2006). Using thematic analysis in psychology. Qual Res Psychol, 3, 77-101.

***Objectives:** Clearly state the primary and secondary objective(s) of the study.*

The primary objective is to examine the feasibility, efficacy, and cost-effectiveness of a mobile-phone-based incentives intervention for promoting smoking cessation among economically disadvantaged (i.e., Medicaid insured) pregnant women.

The secondary objective is to evaluate the acceptability of the treatment, including barriers to and facilitators of treatment engagement.

METHODS AND PROCEDURES

Study Design: *Describe the research design, including a description of any new methodology and its advantage over existing methodologies.*

We are proposing a two condition, parallel groups, randomized controlled trial (RCT) of a mobile-phone-based financial incentives intervention targeting economically disadvantaged pregnant cigarette smokers. The experimental group will receive electronic vouchers contingent on the remote submission of breath carbon monoxide (CO) samples indicating smoking abstinence whereas the control group will receive best practices for promoting smoking cessation. The use of a best practices control group reflects a real-world comparison condition in that all women will receive the treatment that practitioners in the community are instructed to provide (i.e., the 5As plus quit-line referral) thereby enhancing the ecological validity of the study, while also minimizing between-subject variability in the extent of participants' exposure to these practices by implementing these treatment components ourselves (described in greater detail below). Although one common alternative is to use a non-contingent incentives control group which equates both groups in terms of overall earnings, a meta-analysis conducted by our group showed that non-contingent incentives have no impact on abstinence levels above no-incentive control conditions.[44] Alongside the two condition, parallel groups RCT described above, we will recruit an additional 60 American Indian/Alaska Native (AI/AN) women at another site (Alaska Native Tribal Health Consortium, Anchorage, Alaska) to examine effects of the present mobile-phone-based incentives intervention among this specific subgroup of women. Although this smaller subset of women will be randomized separately within an arm that is separate from the main trial, all study protocols and procedures will be identical in the AI/AN arm relative to the main trial.. The rationale for a separate arm targeting AI/AN women is that smoking prevalence is substantially higher among this subpopulation (i.e., ~ 36% for AN women (Patten et al. 2018) versus ~ 13% among U.S. pregnant women overall (Kurti et al., 2017), they exhibit unique tobacco use characteristics including use of a homemade smokeless tobacco (Iqmik, Hurt et al., 2009), and very few smoking cessation interventions have been implemented specifically among AI/AN women (Patten et al., 2010; Patten et al., 2018). Thus, the present smartphone-based incentives intervention—which has never been implemented previously among this subpopulation—will be examined among a subset of 60 AI/AN women. AI/AN women will be recruited at a separate site (ANTHC, Anchorage, AK), and separate IRB approvals will be obtained as mandated by the collaborating location.

Procedures: *Describe all procedures (sequentially) to which human participants will be subjected. Identify all procedures that are considered experimental and/or procedures performed exclusively for research purposes. Describe the types, frequency and duration of tests, study visits, interviews, questionnaires, etc. Include required screening procedures performed before enrollment and while on study. Please provide in table, list or outline format for ease of review. (describe and attach all instruments)*

Note: A clinical research protocol may involve interventions that are strictly experimental or it may involve some aspect of research (e.g., randomization among standard treatments for collection and analysis of routine clinical data for research purposes). It is important for this section to distinguish between interventions that are experimental and/or carried out for research purposes versus those procedures that are considered standard therapy. In addition, routine procedures performed solely for research purposes (e.g., additional diagnostic/follow-up tests) should be identified.

Participants Study participants will be 152 pregnant Medicaid recipients $\geq$ 18 years of age and their infants (304 subjects total). Mom's will be recruited from obstetric practices and WIC offices located in all Vermont counties, and from obstetric practices and WIC offices outside Vermont. We will use the same procedures to recruit as our prior and ongoing trial,[45-48] as well as the print and online advertising strategies (e.g., local newspapers, Facebook, Craigslist) that were developed for use in our current trial. An additional, smaller subset of 60 pregnant AI/AN women will be recruited using a combination of these two methods in Alaska. Briefly, pregnant women attending their first prenatal care visit will be identified from the obstetric clinic's appointment schedules. Receptionists at each practice will request that these women complete a brief self-administered smoking-screening form containing a multiple-choice question on smoking status that has been validated to enhance accurate reporting.[84] These forms will be returned via fax to the Substance Abuse Treatment Center (Room 1415, Arnold Hall). Only those identified as key personnel on the proposed study have access to the fax machine in the SATC. Women who endorse smoking in the 7 days prior to completing the screening form and provide contact information will be contacted by research staff to further verify study eligibility and provide additional information about the study. Regarding our print and online advertising strategies, participants will self-refer after seeing our ads and will complete a screening online or over the telephone with research staff. Prior to enrolling in the study, women who self-refer will be asked to sign a medical release form allowing us to contact their healthcare provider or local WIC director to verify their current pregnancy status.

For inclusion in the study, women must meet the following criteria: (a) $\geq$ 18 years of age, (b) report being smokers at the time they learned of the current pregnancy, (c) report smoking in the 7 days prior to completing their preliminary eligibility screening, (c) $\leq$ 25 weeks pregnant, (d) speak English, (e) own a smartphone (Android or iOS; 81.8% of pregnant women in wave 1 [2013-2014] of the Population Assessment of Tobacco and Health

CHRMS (Medical) #17-0604 Approved: 4/3/2020

[PATH] reported owning a smartphone). Exclusion criteria include: (a) current or prior mental or medical condition that may interfere with study participation (assessed via self-report during formal intake assessment completed online or by phone using a medical and psychosocial history questionnaire), (b) smoke marijuana more than once each week and not willing to quit (marijuana smoking can inflate breath CO), (c) exposed to unavoidable occupational sources of CO (e.g., car mechanic), (d) report currently receiving opioid maintenance therapy (e.g., methadone, buprenorphine). Women who meet the inclusion criteria and complete the informed consent process will be considered formally enrolled and will be mailed equipment to participate in the study, but will not be randomized until they (1) confirm receipt of their equipment, (2) complete an orientation session on the smartphone app used in the current study (described subsequently), and (3) provide a saliva sample indicating current smoking (i.e., test strip returns a positive for cotinine, a metabolite of nicotine). Upon completing these steps, women will be randomized to either the incentives or best practices treatment condition (See Treatment Conditions). The only criteria for withdrawing participants after randomization occurs will be pregnancy termination or fetal demise.

General Study Procedures.

Orientation Session: After verifying eligibility and completing the informed consent process, participants will be mailed two saliva test kits. Upon receiving the saliva tests, participants will be trained in the operation of the smartphone application ("app") used to submit breath and saliva samples remotely and will have the opportunity to practice submitting saliva samples. The first sample that subjects submit upon receiving their saliva test kits will be used to validate smoking status and subjects will be withdrawn prior to randomization if they are found to be non-smokers. Women who provide a baseline saliva test validating that they are smokers will be randomized to one of the two treatment conditions, and will be provided with information about that condition and a brief quiz (see attached) as part of their orientation session. The orientation session will be conducted by telephone. Prior to the orientation session, participants will be mailed a CO monitor, additional saliva cotinine test kits, and an instruction manual containing general information about study procedures and where to download the app that they will use during their participation, as well as specific information about how frequently they should submit breath and saliva samples and a schedule of potential earnings. Given that over 80% of pregnant women can be expected to own smartphones, these women will use their existing phone during their participation to increase the ecological validity of using a mobile phone to deliver the intervention. Women who do not own a smartphone but have access to a computer with Internet access may pursue this option instead. We will collect data on the number of participants who use mobile phones vs. computers to participate in the intervention. During the orientation session, participants will also be informed that we will cover the costs of study-related data transfer if necessary. The purpose of this is to enhance the internal validity of this efficacy study by reducing between subject variability in data coverage. We will also collect data on the number of participants who require assistance paying for data coverage. Other details covered during orientation include instructions specific to the condition to which participants are randomized (e.g., schedule for submitting breath and saliva samples, the schedule of potential earnings, and when the earnings commence). Researchers will read participants an information sheet specific to their treatment assignment (attached), which will be followed by a brief quiz (attached). The purpose of the quiz is simply to verify participant understanding of the condition to which they are randomized. Participants will respond aloud to the quiz questions over the phone and any incorrect answers will be discussed, and they will be invited to ask any additional questions upon completing the quiz. Participants will select a quit date during orientation (either the first Monday following the call or the next Monday), and research staff will contact them by phone on the Friday prior to their quit date. Staff will also inform participants to contact them in the event that their phone is lost, stolen, or broken. In sum, participants who complete the informed consent process will be mailed two saliva test kits to verify their smoking status prior to being assigned to one of the two treatment conditions. Two tests will be sent in case participants' first sample is invalid (e.g., insufficient saliva to produce a reading) and they need to complete a second test to verify smoking status. Participants who are verified to be smokers will then complete the treatment assignment phone call, after which study staff will provide them with the equipment that they will need for the remainder of the study (e.g., breath CO monitor and additional saliva test kits).

Mobile-Phone Based Financial Incentives: The intervention will be delivered on participants' smartphones via an app installed on their phone either before or during the orientation session. The process of submitting a video entails the following steps: (1) Participant opens the app, which requires them to type in a password. Their username and password (stored as a secure, "irreversible" one-way hash) will be stored in a configuration file accessible only to the application; (2) The app will verify the password and participants will be taken to the "home" screen which will show their cumulative earnings to date and a "post video" button; (3) The app will attempt to contact the server over the Internet (via a 3G/4G mobile network) to determine the correct time and status of the participant; (4) The participant will click on the post video button, thereby leading them to a simple interface for recording videos that contains a start/stop recording toggle button, a play button to review the recorded video, and a post button to send the video to the server; (5) After posting the video, the server will display a voucher based on the voucher schedule cached from the prior video upload. The app will then create a text file containing a timestamp and video file. These files will be archived, compressed, and encrypted to prevent tampering and/or eavesdropping while in transit. The app will maintain a file lock on the video until it is discarded

CHRMS (Medical) #17-0604 Approved: 4/3/2020

or posted to prevent participants from tampering with their videos; (6) If a 3G/4G or WiFi Internet connection is available, the app will poll connection status in the background until an upload can be initiated; (7) When the server receives the upload, the video will be extracted and the content registered with the system; (8) A text message verifying that the video was received will be sent to the participant and their account will be updated; (9) Research staff will review and validate videos (see Validating Videos below). Participants will be able to check their recent and cumulative earnings on a mobile-friendly site. These same steps will be employed among participants using the computer-based treatment delivery platform. The app for use in the current study will be designed by DynamiCare Health, Inc. upon receiving the grant award. Eric Gastfriend (Co-Founder/CEO) at DynamiCare Health has received specifications for the design of the app that are identical to what we describe above, and will modify their existing treatment delivery platform to be consistent with these specifications.

Abstinence Criterion: Consistent with our current ongoing clinical trial (CHRMS 13-143), participants will receive incentives during week 1 for all breath CO samples where CO $\leq$ 6 ppm. Breath CO has a relatively short half-life, thus twice daily CO testing during week 1 will help detect recent smoking. This frequency of testing also offers the advantage of allowing women to obtain frequent access to reinforcement thereby engaging them early in the intervention. Although recent studies to promote smoking cessation among pregnant women have employed cut points as high as 10 ppm[87], existing data and our group's experience suggest that moderate levels of smoking can go undetected when using higher cut points. After week 1, incentives will be based on salivary cotinine levels. Salivary cotinine has a longer half-life and thus is more appropriate for the less frequent schedule of routine smoking monitoring that will ensue following week 1. The test itself simply displays a positive or negative, however the equipment specifications indicate that salivary cotinine $\geq$ 30 ng/mL will register as positive which is consistent with the cut point used in prior research conducted by our group.[45]

Participants will be informed during their orientation session about environmental sources that could elevate breath CO, as well as other sources of nicotine that could elevate salivary cotinine. Specifically, research staff will inform participants that they should avoid second-hand or environmental smoke, as well as smoking other combustible tobacco products and/or using marijuana. Thus CO readings above 6 ppm will always be considered positive during week 1 of treatment (described subsequently). Similarly, participants will be informed that other sources of nicotine (e.g., e-cigarettes, nicotine replacement therapy) may result in positive saliva cotinine tests which may prevent them from earning incentives from week 2 onwards.

Smoking Monitoring: We currently plan to use the iCO™ Smokerlyzer® (coVita, Inc.), a handheld Smartphone-compatible CO monitor that connects to the phone using the earphone connector, to monitor smoking status during week 1. Although the iCO™ Smokerlyzer® readings can be viewed using the iCO Smokerlyer® app that is freely available at both the Apple iOS App Store and Google Play Android App Store, we will interface the monitor with our own customized app to permit researchers to validate that breath CO samples are submitted by the intended participants who are enrolled in the study. The iCO™ Smokerlyzer® permits a concentration range of 0-100 ppm and sensitivity results in individual 1 ppm increments. The operating life is approximately 200 tests or 3 years (whichever comes first), which will be adequate for the proposed study. Following week 1, we will use Alere iScreen OFD Oral Cotinine Screening tests to monitor smoking status via saliva cotinine testing. Salivary cotinine has a longer half-life than breath CO, making it a more appropriate measure with less frequent testing. Subjects will submit videos of themselves completing the tests, with each test taking approximately 5 minutes (i.e., 2-3 minutes of swabbing the mouth and tongue, and up to 3 minutes to produce a result). The display indicates whether the sample is either positive or negative, with a positive test registering for salivary cotinine levels $\geq$ 30 ng/mL.

Treatment Conditions.

Best Practices: The 2008 Clinical Practice Guidelines for smoking cessation recommends that pregnant smokers be provided with the 5As.[88] Briefly, these guidelines stipulate that practitioners should implement the following steps at obstetric visits: (1) Ask about smoking status at the first prenatal care visit; (2) Advise those who endorse smoking about the potential harms of smoking to mother and fetus and recommend quitting; (3) Assess the willingness of smokers to make a quit attempt during pregnancy; (4) Assist those willing to make a quit attempt by helping to establish a quit plan, referring them to the Vermont pregnancy-specific quit line and offering assistance with making the initial contact, and by providing them with a copy of the pregnancy-tailored self-help guide "Need Help Putting Out That Cigarette?", distributed by the American College of Obstetricians and Gynecologists; (5) Arrange for follow-up contacts on smoking at subsequent prenatal care visits. As there may be differences in the extent to which the 5As are implemented across obstetric clinics, research staff will implement the 5As at three assessments that take place during pregnancy (see Assessment Procedures below) to decrease between-subject variability in exposure to best practice guidelines. At the first antepartum assessment, staff will complete a fax referral form for participants to the Vermont (or other state) pregnancy-specific quit line, operated by Free & Clear, which provides 9 free telephone-counseling calls with a trained smoking-cessation coach during pregnancy (5 calls) and postpartum (after birth) (4 calls). All calls average approximately 10 minutes. The quit line attempts to reach women five times within a 3-day period for each scheduled phone counseling session. In addition to completing the 5As and referring women to the quit line, all women in the best practices condition will also receive the smoking cessation advice that is provided at their obstetric clinic. Note that pregnant women

CHRMS (Medical) #17-0604 Approved: 4/3/2020

seeking smoking cessation treatment in the community often do not receive cessation-focused follow-up visits after endorsing that they are current cigarette smokers, nor do providers or community health workers typically submit referrals for them to a quit line. As we take these extra steps, we refer to this condition as "best practices" rather than "usual care."

Best Practices + Financial Incentives: Women assigned to this condition will receive the best practices treatment described above plus the remote incentives intervention. As mentioned previously, participants will set a quit date during their orientation session for either the first or second Monday following the session. They may practice submitting samples (for which staff will provide feedback) prior to their quit date if desired, however this is not required as they would have already provided an initial sample to validate their smoking status prior to being randomized. Once the quit date arrives, participants will submit two breath CO samples each day, separated by 8 hours from one another. A day will start at 5:00 a.m. and end at 4:00 a.m. (EST). The app will indicate to participants when samples can be collected because the "post video" button will be locked for 8 hours after the first sample of the day is submitted.

Beginning on the quit date and extending for one week, participants will be required to submit twice daily CO samples. All samples $\leq$ 6 ppm will be considered negative and those > 6 ppm will be considered positive. Requiring frequent testing during week 1 only will permit participants the opportunity to earn frequent reinforcement while their salivary cotinine levels decrease more gradually if participants are not smoking over the course of week 1 of treatment. As with our prior and ongoing trials, the value of the incentive will increase with consecutive negative samples indicating smoking abstinence. If a sample has not been submitted within the specified 8-hour time window, an electronic prompt will be sent to submit a video two hours before the time window expires. Missed samples will be considered positive unless extenuating circumstances are reported (e.g., lost, stolen, broken phone). The schedule of potential earnings will be consistent with our prior trials adjusting for inflation. Thus rather than a maximum potential earnings of $1,200 (2002 USD), participants in the proposed study may earn up to $1,620 (2017 equivalent of $1,200 in 2002) for sustaining smoking abstinence during pregnancy and for 12-weeks postpartum. The schedule of potential earnings will start at $6.25 for the first negative sample and increase by $1.00 for each consecutive negative sample. Thus the second negative sample will be worth $7.25, the third worth $8.25, and so on, until incentive values plateau at a maximum of $33.25. If a participant submits a breath CO > 6 ppm during week 1 or a positive saliva cotinine test any time after week 1, the value of the incentive will be reset to the initial value of $6.25. This reset component is critical to protect against relapse once an initial period of abstinence has been achieved.[89]

Following the initial quit week during which participants submit twice daily breath CO samples, the schedule of monitoring will be reduced and saliva cotinine will be used to determine smoking status. Only tests where the display indicates negative samples will result in subjects earning reinforcement. Specifically, during weeks two through six, participants will submit videos of themselves completing saliva cotinine tests twice per week, then once per week from week seven until delivery. During the once per week phase, the specific day on which participants are required to submit a sample will be determined quasi-randomly (at least two days apart). The sample will be prompted electronically via a text message at the start of the day and the participant will have up to eight hours to provide the sample. This provides a balance between a schedule that is sufficiently unpredictable that participants who are no longer abstinent may be detected, while at the same time providing them with a reasonable time frame to submit a sample upon being prompted to do so. The schedule of potential earnings during weeks two until delivery will be a continuation of the escalating pay schedule from the initial quit week. Importantly, intermittent reinforcement schedules induce more persistence than frequent, predictable schedules,[90,91] thus the schedule we are proposing may be optimal for promoting persistence and sustained abstinence.

As women who quit smoking during pregnancy are particularly vulnerable to relapse during the early postpartum, the frequency of monitoring will increase to twice per week for the initial four weeks postpartum. After the first month, monitoring will be returned to the weekly, quasi-random prompted monitoring schedule for the next 8 weeks. The opportunity to earn incentives will be terminated at the end of postpartum week 12, consistent with our prior and ongoing trials[45,48] thereby permitting us to compare the results of the proposed intervention to our group's traditional, in person financial incentives interventions.

Validating Videos. Research staff will validate participant videos daily during the workweek. After logging in, the process takes less than ten minutes per video. To be considered valid, the videos of breath tests during week 1 must meet the following criteria: (a) have an authentic user (i.e., a known, enrolled participant), (b) participant can be seen holding her breath for the required duration, (d) participant can be seen and heard exhaling into the mouthpiece, and (e) participant displays CO reading at the end of the video until the monitor indicates that the reading is complete. In Dr. Dallery's recent controlled trial that required remote submission of breath CO samples, only 39 of 4,774 (0.8%) total samples submitted were problematic.[55] Of these 39, 35 involved technical issues and four involved discrepancies between the user input and the video-confirmed CO reading. None of the discrepancies resulted in vouchers being provided erroneously. Regarding videos of saliva cotinine testing from week 2 through 12 weeks postpartum, videos must meet the following criteria: (a) have an authentic user (i.e., a known, enrolled participant), (b) saliva cotinine test kit is in view for entire duration of the video, (c)

CHRMS (Medical) #17-0604 Approved: 4/3/2020

participant permits ample time to collect the sample and collect a reading, and (d) test result is displayed clearly at the end of video.

Delivering Incentives. Participants' account activity box on their homepage will display their recent and cumulative earnings. In our group's previous studies that used similar schedules of potential earnings to what we are proposing here, participants earned (on average) $550 during the intervention.[15] Participants in the proposed study will receive a True Link or PEX Debit Card at the beginning of the intervention onto which incentive payments will be loaded contingent on their submission of breath CO samples indicating smoking abstinence. These debit cards do not require a credit history check, and Vermont residents with these cards pay no monthly fees or ATM withdrawal fees at non-MoneyPass ATMs. Moreover there are no costs for using the True Link or PEX Debit Cards for in-store or online shopping. Thus we anticipate that few participants will object to receiving and using these cards. Research staff will load money onto participants' study debit card after reviewing each video and determining that the video meets the criteria for validation above. Comparable methods of incentive delivery have been employed in computer-based financial incentives treatments targeting the general population of smokers.[52-54] In these studies, participants redeemed their incentive earnings via online vendors (e.g., Amazon.com) or had their earnings transferred to a PayPal account after their breath CO submissions were validated. Providing participants with a debit card that is specifically and explicitly for study purposes eliminates the requirement on their part to open a PayPal account, which was unattractive to some participants perhaps because they were not comfortable providing their banking account information to open the PayPal account. Importantly, in-person financial incentives treatments typically require research staff to venture into the community to make a voucher purchase (e.g., gift card) in person and then deliver it to the participant. In contrast, the technology-based method that we are proposing is easy to implement and will decrease both staff travel time to redeem vouchers, as well as the immediacy between engaging in the target behavior and receiving reinforcement.

For research involving survey, questionnaires, etc.: *Describe the setting and the mode of administering the instrument and the provisions for maintaining privacy and confidentiality. Include the duration, intervals of administration, and overall length of participation. (describe and attach all instruments)*

☐ ***Not applicable***

Assessment Procedures. Participants in both the incentives and best practices conditions will complete a formal assessment at intake, during early pregnancy (i.e., one month after enrolling), late pregnancy (i.e., 28-weeks gestation), and at 4-, 8-, 12-, and 24-weeks postpartum. This schedule of formal assessments and the use of similar questionnaires (see Assessment Battery) will permit comparisons to prior controlled trials conducted by our group.[15,45-48] Participants will receive $50 per formal assessment completed. This money may come in the form of a check mailed to their home or in the form of a gift card (TangoCard) that is emailed to them. TangoCard is an electronic gift card platform. If participants receive their rewards via this option, the reward will be emailed to them as a unique URL. By following the URL, the participant can choose from a variety of gift card brands and redeem their reward. Once they select a brand, the final gift card will be emailed to them. To ensure that HIPAA security standards are met and emails are kept private, TangoCard has signed a BAA (Business Associate Agreement) with DynamiCare. Questionnaires will be completed remotely via computer or mobile phone. Items will be administered using REDCap or SurveyGizmo, both of which capture and house unique de-identified codes for study participants. Data audits will be conducted monthly during the first six months of the study and then quarterly until study completion to detect problems. As no personal identifying information will be collected, the chance of a breach of confidentiality is very low. Regardless of whether participants complete the assessment online or over the phone, participants in both treatment conditions will be required to submit both a breath CO sample and a salivary cotinine sample at each formal assessment to determine their smoking status.

Assessment Battery Participant's intake assessment will be conducted after they have had their first prenatal visit and phone screen to determine preliminary eligibility and will address six areas: (a) Sociodemographics (age, educational attainment, race/ethnicity, marital status, health insurance status); (b) Medical/pregnancy history (height/weight, self-reported pre-pregnancy weight, weeks pregnant, history of complications in prior pregnancies; (c) Smoking history (age started smoking, pre-pregnancy time to first cigarette/cigarettes per day, past week time to first cigarette/cigarettes per day, number of quit attempts before/during the current pregnancy, number of other smokers in the household, rules about smoking in the household, nicotine dependence[92]); (d) Smoking timeline follow-back (to characterize daily smoking rates and/or use of alternative tobacco products or nicotine replacement therapy since learning of the current pregnancy[93]); (e) Smoking attitudes (motivation to stop, confidence in ability to stop, intention to quit before the baby is born, intention to remain abstinent after the baby is born, perceived stress levels); (e) Maternal health/executive functioning (lifetime history of depression, general psychiatric symptoms,[94] current depressive symptoms,[95] discounting of delayed hypothetical monetary rewards,[96] behavioral economic measure of the reinforcing value of cigarettes,[68,97] Behavior Rating Inventory of Executive Function®-Adult Form [BRIEF-A; see attached], EQ-5D Health-Related Quality of Life Questionnaire [see attached]); (f) Stressful life events (SAMHSA Life Events Checklist [see attached]). Appropriately modified versions of these measures will be administered at the other six formal assessments identified above. In addition, at the postpartum assessments we will assess breastfeeding, including initiation, duration, and different levels of breastfeeding (e.g., exclusive, predominant, any).

CHRMS (Medical) #17-0604 Approved: 4/3/2020

Participants randomized to the Incentives condition will have one additional measure administered at five of the six formal assessments that follow the intake assessment (early pregnancy, late pregnancy, 4-, 8-, and 12-weeks postpartum). This measure is an Episodic Future Thinking task (EFT; see attached) that requires them to imagine positive events that may realistically occur at various time points in the future. Participants will answer open-ended questions asking them to describe the event, who will be present, how they will feel, etc. They will also rate the event on a series of characteristics such as how positive, vivid, and detailed the future event is. The time points at which participants are asked to imagine future events will correspond to subsequent assessments (e.g., participants completing their early pregnancy assessment will be asked to imagine an event that may realistically occur by the time they reach their late pregnancy assessment). Instructions provided before and after the text explain the reason and potential benefits of engaging in EFT, and encourage participants to think about the cues that they generate in between assessments (e.g., first thing in the morning to set their day, when they experience cravings or feel tempted to smoke).

After each assessment is complete, research assistants will review participants' responses to the EFT questions, and may provide feedback about how participants can use their future event in their daily lives as motivation to quit smoking or to stay smoke-free. Participants may also receive notifications from the study app prompting them to think about their future events in between their formal assessments. The rationale for adding this component to the Incentives group only is that engaging in EFT may help these women sustain abstinence more effectively after the incentives are withdrawn at 12 weeks postpartum. Instructions and prompts to think about their future events and incorporate these cues into their daily lives further encourages participants to practice prospection, and thus may be helpful in better sustaining long-term abstinence. Any texts that participants receive asking them to think about their future events will be automatically generated from the DynamiCare Rewards App. Since these messages will be auto-generated from the study app, they will not address the participants by name, and the content will not be specific to individual participants in the study, nor will the content include any personalized messaging or identifying information.

After participants' consent to join the study, we will collect contact information over the phone including their mailing address (to send them equipment), as well as the phone number for an alternative contact in the case that we are unable to reach the participant. We will also ask the participant to tell us how we should describe their involvement in the research study should we need to reach out to their alternative contact at some point. This form is attached, and it will be updated following each formal assessment. Keeping an updated mailing address is important in case participants need additional equipment or paperwork mailed to them.

We also plan to examine birth outcomes and their associated costs in order to gather basic data for a preliminary cost-effectiveness analysis. More specifically, project staff will obtain faxed copies of the delivery reports for all women enrolled in the study. Mom's verbal permission allowing researchers to recruit and collect medical record data about their infant will be documented during the informed consent process. For infants born at UVMMC, this will constitute permission to access infant health outcomes in PRISM. For infants born elsewhere, Mom's may be mailed a Medical Release Form (attached) after delivering their baby that they will sign and return in a stamped envelope pre-addressed to the UVM SATC (Room 1415, Arnold Hall), or they may view this medical release form and provide an electronic signature on RedCAP if the location where they deliver accepts e-signatures as a valid form of authorization. Regardless of whether signatures on the medical release form are obtained electronically or via the mail,this form will then be faxed to the clinics where they delivered. We use comparable methods to obtain infant medical record data in our current trial (CHRMS 13-143) and have received a vast majority of delivery reports for infants born outside UVMMC. Outcomes abstracted from the delivery report will include mean birth weight, % low birth weight deliveries (< 2500 g), mean gestational age at delivery, % premature deliveries (< 37 weeks), incidence of NICU admissions, and mean length of stay per NICU admission. Over the past 10 years, over 99% of women in our trials have provided permission to review maternal and infant medical records, so we anticipate no difficulty obtaining consent to collect these data in the proposed study. Our staff has substantial experience establishing relationships with local primary care clinicians, which facilitates the logistics of our staff coordinating with new clinics to review records.

Regarding the cost-effectiveness analysis, we will employ the Brief Drug Abuse Treatment Cost Analysis Program (Brief DATCAP;[98,99]) to estimate the cost of delivering the Incentives versus Best Practices smoking cessation treatments. More specifically, we will derive the direct and indirect economic cost of treatment by allocating fixed costs based upon the proportion of time spent delivering these programs, as well as costs that vary by patient engagement and smoking status (e.g., staff time validating breath CO samples, quit-line staff time, incentives). Administration costs (e.g., postage/courier service to mail participants CO monitors) will also be included. Costs will be in USD for price year 2017/2018. The time period of the cost analysis will span from intake to discontinuation or completion of the program. However, since the duration of treatment will vary according to where in the pregnancy a woman enters the study, the economic cost per person per week will also be calculated. Research-specific resources consumed over the course of the study will be excluded from the cost-effectiveness analysis. Estimated treatment delivery costs will be combined with the delivery report outcomes above to conduct the preliminary economic evaluation (See Statistical Methods).

Examining Acceptability. At the 24-week postpartum assessment or shortly thereafter, participants in both

CHRMS (Medical) #17-0604 Approved: 4/3/2020

the incentives and usual care conditions will complete a Treatment Acceptability Questionnaire (TAQ) comparable to those administered in other financial incentives interventions,[21] including research conducted by Dr. Kurti.[61,64] The measure will be administered on REDCap and will query participants about the ease of use, helpfulness, and convenience of the intervention, as well as whether the intervention was fair, fun, and whether they would recommend it. All responses will be made using a 100-point visual analogue scale. Participants in the incentives condition will receive additional TAQ questions inquiring about whether they liked self-monitoring their breath CO levels, engaging in EFT, earning vouchers, and whether the EFT tasks and the vouchers were helpful in terms of promoting smoking abstinence during treatment and sustaining abstinence following treatment withdrawal. In addition, research staff will call all participants upon completing the 24-week assessment/TAQ to query them about barriers and facilitators of treatment engagement including: (a) features of the intervention that they felt facilitated or hindered engagement (e.g., technical difficulties/availability of technical support, self-monitoring using the CO monitor, appropriateness of staff counseling surrounding smoking), (b) social/environmental variables (e.g., quit support from friends/family, rules about smoking in the home, exposure to smoke-free environments, social networks that promoted or discouraged quitting smoking) and (c) internal/psychological variables (e.g., stress, psychological well-being, motivation to quit, self-efficacy for quitting smoking). The semi-structured interview used to examine barriers and facilitators of treatment engagement will use scripted prompts, however participants will be able to respond freely and openly to each question. Their responses will be transcribed verbatim by the research staff completing the interviews.

Statistical Considerations: *Delineate the precise outcomes to be measured and analyzed. Describe how these results will be measured and statistically analyzed. Delineate methods used to estimate the required number of subjects. Describe power calculations if the study involves comparisons. Perform this analysis on each of the primary and secondary objectives, if possible.*

Statistical Methods

Study conditions will be compared on baseline demographics and other characteristics using analysis of variance (ANOVA) for continuous variables and chi-square tests for categorical variables. If a specific characteristic differs significantly across study conditions and is predictive of the outcome, it will be considered as a potential covariate in subsequent analyses. Analyses of treatment effects on smoking status will adhere to an intent-to-treat approach[100] whereby all women randomized to each study condition will be included in the analyses independent of early dropout, noncompliance, etc., with the exception of women excluded for abortion/fetal demise as is convention in this research area. Cochran-Mantel-Haenszel tests (C-M-H) will be performed for comparisons between the incentives vs. usual care conditions on point-prevalence smoking abstinence at end-of-pregnancy and 6-months postpartum assessments with referring clinic as a stratification variable. The Breslow-Day Test will be used to examine the homogeneity of treatment effects across referring clinics. Comparisons of point-prevalence abstinence rates between treatment conditions across all assessments through six months postpartum will be analyzed using mixed model repeated measures for categorical data based on generalized estimating equations (GEE) using a logistic link function (SAS: PROC GENMOD, SAS Institute, Cary, NC). The two treatment groups will also be compared on birth outcomes, breastfeeding, and other measures collected postpartum. Comparisons of treatment conditions on dichotomous outcomes (e.g., % low birth weight, % preterm, % NICU admissions, % breastfeeding) will parallel categorical analyses for point prevalence abstinence using PROC GENMOD to adjust for strata (referring clinics) and potential covariate effects. Analysis of covariance (ANCOVA) will be used to compare study conditions on mean birth weight adjusting for variables known to influence birth weight (% maternal pre-pregnancy BMI, parity, and sex) and mean gestational age at delivery. The significance criterion will be set at alpha = 0.05 for all analyses.

Regarding the cost-effectiveness analysis (CEA), the CEA will be conducted by dividing the average (mean) difference in treatment costs across the incentives versus best practices conditions by the average (mean) difference in each outcome (abstinence rates in late pregnancy, birth outcomes) to derive incremental cost-effectiveness ratios (ICERs).[101] Statistical significance of these ICERs will be determined probabilistically by employing non-parametric bootstrapped standard errors.[102] The main objectives of this preliminary CEA will be to (a) establish methods for obtaining and analyzing data that pertain to both treatment delivery and birth outcomes in remote financial incentives interventions, and (b) comparing ratios on key birth outcome measures and NICU admissions. These basic CEA data will provide important preliminary data for a future RO1 application in which we may propose conducting a more sophisticated cost-effectiveness analysis that incorporates both birth outcomes and infant health outcomes assessed over a longer time period (e.g., medical illnesses throughout the infant's first year of life).

Regarding the acceptability data, TAQ items administered using a 100-point VAS will be compared across the two treatment conditions using ANOVA's. Again characteristics that differ across treatment condition will be considered as potential covariates. Responses to the semi-structured interview questions will be transcribed directly during the interview and analyzed afterwards using a thematic content analysis.[103,104] We will employ an inductive approach whereby the content of these qualitative data will direct the coding and theme development. Coding of the data will involve grouping, sorting, and identifying themes that reflect barriers and facilitators of treatment engagement. Two researchers will perform the content analysis independently and sort responses into

CHRMS (Medical) #17-0604 Approved: 4/3/2020

themes. Disagreements will be resolved through discussion until consensus is reached. Those themes that emerge and reflect modifiable aspects of the intervention (e.g., provision of technical support, appropriateness of staff counseling and feedback surrounding quitting smoking) will be taken into account in designing future iterations of this intervention that are more responsive to participant's preferences.

Comparable analyses may be conducted among the smaller subset of AI/AN pregnant women. However, as financial-incentives-based interventions to promote smoking cessation have never previously been examined among this subgroup using either in-person or remote methods of treatment delivery, this additional subset of pregnant women is more exploratory at this stage, and provides an important opportunity to conduct a feasibility and proof of concept assessment. As our examination of the intervention among a subgroup of AI/AN women represents the first feasibility and proof of concept assessment to our knowledge, power analyses were not conducted to determine the sample size needed to obtain statistically significant effects on the above outcomes. However, once we complete our assessment of the feasibility and efficacy of smartphone-based financial incentives among this subset of AI/AN women, such analyses will be conducted to determine the sample size needed in future, larger-scale studies targeting this unique subpopulation.

Sample Size Justification

Based on data from (a) our prior and ongoing trials which utilized a comparable schedule of routine smoking monitoring visits and schedule of potential earnings, and (b) previous trial data indicating a 20% decrease in abstinence rates from late pregnancy to 24-weeks postpartum, we expect late-pregnancy abstinence rates of approximately 40% vs. < 10% (incentives vs. best practices, respectively), and 24-week postpartum abstinence rates of approximately 20% vs. < 5%. The proposed sample size of 76 per treatment condition will result in at least 80% power to detect a difference between the two treatment conditions in abstinence rates of 40% vs. 10 % at late-pregnancy or 20% vs. 5% at 24-weeks postpartum assessments using a chi-square test. This proposed sample size is also expected to be more than sufficient for examining differences between treatment conditions in birth weight and breastfeeding outcomes. With respect to mean birth weight, power is estimated to be 60% to detect a difference of approximately 200 g between the incentives versus best practices conditions assuming a pooled standard deviation (SD) of 550 g, which aligns well with treatment effects in our study aggregating birth outcomes across our first three controlled trials[16] whereby mean birth weights were 3295.6 $\pm$ 588 g vs. 3093.6 $\pm$ 404 g in the incentives versus control conditions, respectively. We are estimating breastfeeding rates of 50% and 30% in the incentives condition and 17% and 13% in the best practices condition at 12 and 24-weeks postpartum based on our prior study results.[17] Using chi-square tests, we will have over 75% power to detect differences in breastfeeding rates between the incentives versus best practices conditions at the two assessments. Power calculations were done using a two-sided significance level of 0.05. A sample size of 60 for the arm targeting AI/AN women was selected based on initial pilot studies of the financial-incentives-based approach to promoting smoking cessation among pregnant women developed at the University of Vermont (Higgins et al., 2004). This sample size will be sufficient to determine whether the intervention is feasible and effective at promoting late-pregnancy smoking abstinence between AI/AN women enrolled in the Incentives versus Best Practices Control conditions. Results of our assessment will be used to inform power analysis calculations for larger-scale, NIH-supported research proposals focused on smoking cessation among AI/AI pregnant women.

Risks/Benefits: *Describe any potential or known risks. This includes physical, psychological, social, legal or other risks. Estimate the probability that given risk may occur, its severity and potential reversibility. If the study involves a placebo or washout period, the risks related to these must be addressed in both the protocol and consent. Describe the planned procedures for protecting against or minimizing potential risks and assess their likely effectiveness. Where appropriate, discuss plans for ensuring necessary medical or professional intervention in the event of adverse effects to the subjects. Discuss the potential benefits of the research to the subjects and others. Discuss why the risks to the subjects are reasonable in relation to the anticipated benefits to subjects and others. Discuss the importance of the knowledge gained or to be gained as a result of the proposed research and why the risks are reasonable in relation to the knowledge that reasonably may result. If there are no benefits state so.*

Risks

Participants may experience some discomfort arising from nicotine withdrawal. Participants will be informed during the informed consent process that they may experience the following symptoms of nicotine withdrawal: craving cigarettes, restlessness, irritability, increased appetite, increased eating, dizziness, difficulty concentrating, and depressed mood. There is a small risk that participants' electronic information could be accessed thereby affecting confidentiality. There is a risk that women may be uncomfortable answering some of the questions on the formal assessment batteries. There is a risk that use of other combustible tobacco products and/or marijuana during study participation may elevate participants' breath CO levels thereby preventing them from earning incentives during week 1 even if they have quit smoking cigarettes. Similarly, use of other nicotine products like e-cigarettes or nicotine replacement therapy could elevate salivary cotinine levels after week 1 and prevent subjects from earning incentives. There is also a risk that a participant may become distraught during the course of the study and become a danger to herself, or that other emergencies could arise that research staff may need to address.

Summary of Protection Against Potential Risks

We will take the following actions to protect against potential risks: (a) We will inform women during the informed consent process that reducing and/or quitting smoking may result in nicotine withdrawal symptoms however these symptoms should disappear within two weeks. (b) To ensure participant confidentiality, all study files will be stored in locked filing cabinets. All participants receive a subject identification code that is used in place of their name in all study files. The key connecting names and ID codes is kept in a locked file and stored separately from the data files. Study computers are password protected and encrypted. These protections apply only to study data collected via paper/pencil methods (e.g., phone eligibility screening data recorded by research staff). (c) For subjects who self-refer and complete their preliminary eligibility screening online, the survey will be administered using UVM's REDCap system. REDCap (Research Electronic Data Capture) is a secure, web-based application designed to support data capture for research studies that is hosted at UVM. REDCap has various security features, such as requiring usernames & passwords to access REDCap, and then needing permissions for the individual project. REDCap is accessed by URLs starting with HTTPS which encrypts data before being sent over the internet. (d) With respect to the assessment batteries conducted using REDCap or SurveyGizmo, we will not ask participants to self-report identifying information and both the REDCap and SurveyGizmo platforms are configured such that they do not retain identifying information (including IP addresses), thus a breach of confidentiality is exceedingly unlikely. (e) Participants will be informed during the informed consent process and again before completing the assessment batteries that they may skip questions or stop at any time should they feel uncomfortable answering some of the items. (f) Regarding the use of other tobacco products, we will inform women that the use of other combustible products could influence their breath CO and thereby limit their ability to earn incentives during week 1, and that using other nicotine-containing products following week 1 could influence saliva cotinine levels thereby preventing them from earning incentives from week 2 onwards. (g) If the investigative team or research staff are concerned that a given participant may be experiencing untreated psychiatric, family/social or other problems (e.g., suicidal thought), this information will be immediately acted upon by staff and investigators (e.g., crisis services in the participant's community will be contacted). Research staff will be trained to deal with emergency calls. Protocols for assessing and responding to suicidality include a suicidality assessment checklist and a plan to call Crisis Services if any intent is demonstrated.

The study investigators will oversee all procedures designed to continuously monitor participant safety. The investigative team will meet once each week to discuss actual or potential issues for each participant. During these meetings, investigators will review all participants' progress to ensure treatment fidelity

Benefits

The benefits of the study are considerable. All participants will receive the 5As from research staff as well as the opportunity to have a staff member submit a fax referral form on their behalf to the Vermont (or other state) pregnancy-specific quit line. Thus all participants will receive instructions about when to quit and counseling surrounding their smoking, as well as feedback about their smoking status upon providing breath CO and saliva samples. If they are successful in abstaining from smoking, all participants will receive positive feedback. Importantly, as both conditions involve treatment, the primary benefit of participating will be the potential for women to quit smoking, which may have important immediate and long-term health benefits for both the mother and her offspring. Women randomized to the contingent incentives condition have the opportunity to earn vouchers that can be used to purchase goods and services that may help improve their quality of life (e.g., groceries, gas, baby clothes).

There is a reasonable likelihood that women assigned to the best practices condition will not achieve outcomes as good as those assigned to the best practices plus incentives condition. Nevertheless, all women will receive treatment that meets Best Practices as outlined in the 2008 Clinical Practice Guidelines on Treating Tobacco (Fiore et al., 2008).

Although the study is considered high-risk by definition (i.e., pregnant population), the potential benefits are substantial in terms of our scientific understanding of the effectiveness of mobile-phone-based financial incentives interventions targeting pregnant smokers. Overall, the risk/benefit ratio appears highly favorable.

Importance of the Knowledge to be Gained

Cigarette smoking is the largest preventable risk factor for morbidity and mortality in developed countries and involves considerable risks to fetal and infant health. Smoking during pregnancy can lead to spontaneous abortion, preterm birth, stillbirth, low-birth weight, and sudden infant death syndrome, as well as the development of later in life chronic conditions. The proposed study may suggest that mobile phone based incentives hold significant clinical utility and promise. If so, the platform for delivering the intervention remotely is sufficiently flexible that it could be modified to treat other vulnerable populations such as those with mental illness or adolescents in the future. Because distance and traveling are not limiting factors in applying the treatment, the system may also prove to be especially beneficial among rural populations. In short, the knowledge gained could help us develop an effective and broadly applicable treatment to mitigate the morbidity and mortality associated

with cigarette smoking during pregnancy.

Therapeutic Alternatives: *List the therapeutic alternatives that are reasonably available that may be of benefit to the potential subject and include in the consent form as well.*

☐ ***Not Applicable***

Women can utilize the free State tobacco quit line and related services outside of the context of this study. They can also choose to rely on the services offered by their providers.

Data Safety and Monitoring: *The specific design of a Data and Safety Monitoring Plan (DSMP) for a protocol may vary extensively depending on the potential risks, size, and complexity of the research study. For a minimal risk study, a DSMP could be as simple as a description of the Principal Investigator's plan for monitoring the data and performance of safety reviews or it could be as complex as the initiation of an external, independent Data Safety and Monitoring Board (DSMB). The UVM/UVM Medical Center process for review of adverse events should be included in the DSMP.*

Data Safety and Monitoring Plan

Our overall monitoring plan consists of ongoing, close monitoring of data and safety issues by the PI and other project staff and prompt reporting of any adverse events (AEs) or serious adverse events (SAEs) to the institutional review board and/or NIH.

Patient eligibility and status

All recruitment will be managed by trained research staff under the supervision of the PI using specialized forms and procedures. All information collected will be reviewed by the research staff, PI, or designated representatives, who will determine participant eligibility, contact them about scheduling and completing an intake assessment where appropriate. Eligible women will complete the informed consent process over the phone, after which they will be mailed a paper copy of the full consent form along with their equipment to participate in the study. The consent procedure is described in greater detail elsewhere and recent research published in JAMA demonstrates the feasibility of completing the informed consent process over the phone (McConnell et al., 2017). The status of all active participants will be reviewed weekly at staff meetings between the PI/Research Assistant and other trained support staff.

Confidentiality

Steps will be taken to ensure confidentiality of all written and electronic information. With regard to written information, all study files will be stored in locked filing cabinets. All participants receive a subject identification code that is used in place of their name in all study files. The key connecting names and ID codes is kept in a locked file and stored separately from the data files. Study computers are password protected and encrypted. With respond to electronic information, the server will be protected from outside intrusion by multiple firewalls. Administrative access to the machines will only occur using SSH, a secure, encrypted protocol for remotely connecting to a machine. The servers will be hardened against attack using some of the concepts deployed in the Bastille Linux project, a project whose purpose is to automate some of the processes involved in hardening the Linux operating system. Security will be periodically monitored using network mapping tools, like nmap which probe machines for vulnerabilities and report the results to the system administrator. The software Port Scan Attack Detector (PSAD) will be used to monitor the servers and notify administrators if they come under attack. The Advanced Intrusion Detection Environment (AIDE) will be used to detect if any unauthorized changes are made to the machine, allowing us to identify and fix changes that might be made to critical files in the unlikely event of a break-in. Servers will be backed up nightly to external hard drives using an encrypted file system. External hard drives will be rotated offsite weekly and stored in the PD's office in a locked, data-safe firebox. The software vendors/developers websites will be monitored for security patches and upgrades. Additionally, the server appliance's Linux operating system provides a means for monitoring and regularly installing security patches. See the Data Monitoring Plan for further information about protection against risks.

As staff training is crucial to ensuring confidentiality, all study personnel will receive certification in human subjects' protection from the Collaborative Institutional Training Initiative (CITI) prior to beginning work on this project.

Auditing procedures

Review of any problems related to quality of data collection, transmission or analyses, and of any AEs and SAEs that occurred during the past week will occur at weekly research staff meetings.

Adverse Event and Unanticipated Problem (UAP) Reporting: *Describe how events and UAPs will be evaluated and reported to the IRB. All protocols should specify that, in the absence of more stringent reporting requirements, the guidelines established in the Committees on Human Research "Adverse Event and Unanticipated Problems Reporting Policy" will be followed. The UVM/UVM Medical Center process for review of adverse events and UAPs to subjects or others should be included in the DSMP.*

In the proposed study, we will use the FDA's definition of AEs and SAEs. AEs and SAEs will be assessed at each

CHRMS (Medical) #17-0604 Approved: 4/3/2020

subject visit by a trained staff member, and will be discussed at the weekly research staff meetings. Any SAE will be brought to the attention of the PI as soon as possible and not longer than 24 hours. Any AE or SAE that is both unexpected and related to the study participation will be reported to the IRB within 7 days of the event. That IRB will make a determination as to whether additional reporting requirements are needed. IRB actions will be reported to the funding agency by the PI no less than annually and more frequently as recommended by the local IRB. Any SAEs will be summarized in the yearly Progress Reports to the funding agency, including a review of frequency and severity. All SAEs will be followed through ongoing consultation with the physician caring for the patient until they resolve, result in death, or stabilize and are not expected to improve.

Withdrawal Procedures: *Define the precise criteria for withdrawing subjects from the study. Include a description of study requirements for when a subject withdraws him or herself from the study (if applicable).*

Women will be free to withdraw at any point during the course of the study without penalty. That will be explained during informed consent. In terms of data analysis, we will adhere to an intent-to-treat approach (Armitage, 1983) wherein all women randomized to the study conditions will be included in the analyses independent of early dropout, noncompliance, etc., with the exception of excluding women for abortion/fetal demise as is convention in this research area. Women who receive their equipment and fail to complete an orientation session, as well as women who provide a baseline CO sample indicating that they are non-smokers, will be withdrawn prior to randomization and thus not included in an intent-to-treat analysis.

Sources of Materials: *Identify sources of research material obtained from individually identifiable human subjects in the form of specimens, records or data. Indicate whether the material or data will be obtained specifically for research purposes or whether use will be made of existing specimens, records or data.*

The research materials to be obtained include interviews, questionnaires, breath and saliva specimens to verify smoking status (and serve as the basis for reinforcement in the incentives condition), and medical records to assess birth outcomes. Materials will be collected remotely using a combination of phone calls with participants, online surveys, and remote video capture via smartphone app (i.e., for submission of breath CO samples and salivary cotinine samples). Delivery reports will be obtained via fax and entered into databases with no identifying information (i.e., with subject ID only). These databases are stored on password-protected external hard drives in locked offices in a locked clinic accessible only to the PI and research staff.

DRUG AND DEVICE INFORMATION

Investigators are encouraged to consult the UVM Medical Center Investigational Pharmacy Drug Service (847-4863) prior to finalizing study drug/substance procedures.

Drug (s) [X] ***Not applicable***

Drug name – generic followed by brand name and common abbreviations. Availability – Source and pharmacology; vial or product sizes and supplier. If a placebo will be used, identify its contents and source. (attach investigational drug brochure)

Preparation: Reconstitution instructions; preparation of a sterile product, compounded dosage form; mixing guidelines, including fluid and volume required. Identify who will prepare.

Storage and stability – for both intact and mixed products.

Administration – Describe acceptable routes and methods of administration and any associated risks of administration.

Toxicity – Accurate but concise listings of major toxicities. Rare toxicities, which may be severe, should be included by indicated incidence. Also adverse interactions with other drugs used in the protocol regimen as well as specific foods should be noted. Address significant drug or drug/food interactions in the consent form as well. List all with above details.

Is it FDA approved: (include FDA IND Number)

1. *in the dosage form specified? If no, provide justification for proposed use and source of the study drug in that form.*
2. *for the route of administration specified? If no, provide justification for route and describe the method to accomplish.*
3. *for the intended action?*

Device (s) [X] ***Not applicable***

Device name and indications (attach investigational device brochure)

Is it FDA approved: (include FDA IDE Number)
1. for indication specified? If no, provide justification for proposed use and source of the device.

Risk assessment (non-significant/significant risk) - PI or sponsor needs to assess risk of a device based upon the use of the device with human subjects in a research environment.

SUBJECT CHARACTERISTICS, IDENTIFICATION AND RECRUITMENT

Subject Selection: *Provide rationale for subject selection in terms of the scientific objectives and proposed study design.*

We are studying economically disadvantaged pregnant cigarette smokers (i.e., Medicaid recipients) and their infants to develop more effective interventions to help this population quit smoking. As noted previously, the frequent biochemical verification required by in-person financial incentives interventions often limits the number of individuals we can treat, as some women live in remote areas and/or lack reliable transportation to the clinic. Targeting pregnant smokers in a remotely delivered intervention may provide an innovative means of transcending these historical barriers to treatment. Regarding the smaller subset of 60 AI/AN pregnant women, smoking prevalence is substantially higher among this subpopulation (i.e., ~ 36% for AN women (Patten et al. 2018) versus ~ 13% among U.S. pregnant women overall (Kurti et al., 2017), they exhibit unique tobacco use characteristics including use of a homemade smokeless tobacco (Iqmik, Hurt et al., 2009), and very few smoking cessation interventions have been implemented specifically among AI/AN women (Patten et al., 2010; Patten et al., 2018). Thus, their inclusion represents the first examination of the feasibility and effectiveness of financial incentives to reduce smoking targeting this specific subpopulation, and may provide important preliminary data for future grant applications to disseminate the present intervention among AI/AN women on a larger scale.

Vulnerable Populations: *Explain the rationale for involvement of special classes of subjects, if any. Discuss what procedures or practices will be used in the protocol to minimize their susceptibility to undue influences and unnecessary risk (physical, psychological, etc.).*

☐ ***Not applicable***

Pregnant women and their infants to develop more effective interventions to help them quit smoking and to examine improvements in birth outcomes from doing so.

Number of Subjects: *What is the anticipated number of subjects to be enrolled at UVM/UVM Medical Center and in the case of a multi-center study, with UVM/UVM Medical Center as the lead, the total number of subjects for the entire study.*

152 women and their infants in the main trial plus 60 AI/AN women and their infants recruited at a different site (ANTHC, Anchorage, AK) = 424 subjects total.

Inclusion/Exclusion Criteria: *Eligibility and ineligibility criteria should be specific. Describe how eligibility will be determined and by whom. Changes to the eligibility criteria at a later phase of the research have the potential to invalidate the research.*

A total of 152 Mom's will be recruited for the main trial plus 60 AI/AN women recruited at our collaborating site (ANTHC, Anchorage, AK). Participants will be healthy, socioeconomically disadvantaged (i.e., Medicaid insured) pregnant smokers. The inclusion criteria include: (a) ≥ 18 years of age, (b) report being smokers at the time they learned of the current pregnancy, (c) report smoking in the 7 days prior to completing their phone eligibility screening, (c) ≤ 25 weeks pregnant, (d) speak English, (e) own a smartphone (Android or iOS; 81.8% of pregnant women in wave 1 [2013-2014] of the Population Assessment of Tobacco and Health [PATH] reported owning a smartphone). The exclusion criteria include: (a) current or prior mental or medical condition that may interfere with study participation, (b) smoke marijuana more than once each week and not willing to quit (marijuana smoking can inflate breath CO), (c) exposed to unavoidable occupational sources of CO (e.g., car mechanic), and (d) currently maintained on opioid maintenance therapy. The only criteria for withdrawing someone from the trial following randomization to treatment condition will be pregnancy termination or fetal demise. Women who fail to complete an orientation session or provide an initial CO sample indicating that they are smokers will be withdrawn prior to randomization. Criteria will be assessed during a phone screen to determine preliminary eligibility and further evaluated during the formal intake assessment.

Inclusion of Minorities and Women: *Describe efforts to include minorities and women. If either minorities or women are excluded, include a justification for the exclusion.*

This project will include only women. Smoking among pregnant and newly postpartum women has sufficiently special circumstances regarding the potential toxicity to the fetus and newborn, the reasons for trying to quit smoking and prevent relapse, and the patterns of smoking reduction, cessation, and relapse that they need to be studied exclusively in women.

There will be no exclusion criteria concerning race or ethnicity. In Vermont, minorities comprise 4% of the general population. In our prior NIH studies with pregnant smokers, approximately 6-8% of participants were

CHRMS (Medical) #17-0604 Approved: 4/3/2020

minorities. We shall do all that we can to assure that minorities are included in the research and have set a goal of 10% minority participation in the proposed study.

We will work closely with providers in the communities in which we recruit who have a greater minority representation in their practices to increase awareness of the importance of smoking cessation, especially among pregnant women, and the longstanding and excellent record of our team in conducting ethical studies that advance knowledge while also enhancing the quality of health care that is provided throughout the community, but especially the more economically disadvantaged segments of our community. We have been providing free, effective substance abuse treatment as part of NIH research studies in the more disadvantaged parts of Burlington and surrounding communities continuously for more than 25 years, including starting the first ever specialty clinics for cocaine and opioid dependence.

Also important to underscore is that the results from our seminal studies on the use of financial incentives in treating abuse of cocaine and other illicit drugs were specifically tested for generalization to ethically and geographically diverse samples and shown to generalize without any discernible differences in outcome (Silverman et al., 1996; Petry et al., 2005), leading to eventual international adoption of the treatment approach (Pilling et al., 2007; Secades-Villa et al., 2008). We anticipate similar success with dissemination of this model of smoking cessation for pregnant women to ethically and geographically diverse populations of pregnant smokers.

Inclusion of Children: *Describe efforts to include children. Inclusion is required unless a clear and compelling rationale shows that inclusion is inappropriate with respect to the health of the subjects or that inclusion is inappropriate for the purpose of the study. If children are included, the description of the plan should include a rationale for selecting or excluding a specific age range of children. When included, the plan must also describe the expertise of the investigative team in working with children, the appropriateness of the available facilities to accommodate children, and the inclusion of a sufficient number of children to contribute to a meaningful analysis relative to the purpose of the study.* ***If children are excluded*** *then provide appropriate justification. Provide target accrual for this population.*

As we plan to collect limited health information about the infants born to Mom's enrolled in the study, Mom's will provide permission to recruit and collect the following pieces of information about their infants: birth weight, gestational age at delivery, whether the infant was admitted to the NICU, and duration of NICU stay. Collecting these data are necessary in order to evaluate effects of this novel treatment on infant birth outcomes and to complete the proposed basic cost-effectiveness analysis. Mom's will be informed during the consent process about the specific pieces of information that we plan to collect about their infant, and the reasons for collecting this information.

For protocols including the use of an investigational drug, indicate whether women of childbearing potential have been included and, if not, include appropriate justification.

N/A

If HIV testing is included specifically for research purposes explain how the test results will be protected against unauthorized disclosure. Include if the subjects are to be informed of the test results. If yes, include the process and provision for counseling. If no, a rationale for not informing the subjects should be included.

X ***Not applicable***

Recruitment: *Describe plans for identifying and recruitment of subjects. All recruitment materials (flyers, ads, letters, etc) need to be IRB approved prior to use.*

As in our prior and current trials, applicants will be recruited from obstetrical/WIC clinics and using print and online media advertisements. With regard to the obstetrical clinics, the screener form that we use has been demonstrated to increase disclosure of smoking status among pregnant women. Although the form will be administered by medical assistants and receptionists at the clinics, participants will make a voluntary decision whether to provide their contact information thereby giving the research staff permission to contact them with more information about they study. All recruitment materials are attached. ObGyn clinics/WIC offices will fax these forms to the copy machine in the Substance Abuse Treatment Center (Room 1415, Arnold Hall). There are seven full-time RA's and two postdoctoral fellows in the SATC, all of whom have been identified as key personnel on the proposed study, thus nobody other than KP will have access to the smoking screener forms faxed to us. These same recruitment procedures will be employed to recruit the smaller subset of 60 AI/AN women as well. More specifically, Dr. Kathy Koller, MD and Site Principal Investigator for the collaborating site, will leverage her connections to obstetrical/WIC clinics within and surrounding Anchorage, AK. The same screener form being administered to participants for the main trial will be administered in Alaska, and faxed to the same copy machine in the SATC described above. The only difference in recruitment procedures is that separate Facebook ads targeting AI/AN women may be deployed in Alaska. The text for these ads, as well as exemplars of pictures, are attached to this submission. These ads were developed based on prior research conducted by the ANTHC involving online recruitment of AI/AN women. Although the images and text differ slightly from our currently approved ads for the main trial, the same link to the Redcap preliminary screening items will be displayed on the ads targeting AI/AN women. Thus, after AI/AN women interact with the Facebook ad, all study procedures moving forwards will identical to those described for the main trial participants.

FINANCIAL CONSIDERATIONS

Expense to Subject: *If the investigation involves the possibility of added expense to the subject (longer hospitalization, extra studies, etc.) indicate in detail how this will be handled. In cases where the FDA has authorized the drug or device company to charge the patient for the experimental drug or device,* ***a copy of the authorization letter from the FDA or sponsor must accompany the application. Final approval will not be granted until the IRB receives this documentation.***
There are very limited circumstances under which study participants may be responsible (either directly or via their insurance) for covering some study-related expenses. If the study participant or their insurer(s) will be billed for any portion of the research study, provide a justification as to why this is appropriate and acceptable. For example, if the study involves treatment that is documented standard of care and not investigational, state so. In these cases, the protocol and the consent should clearly define what is standard of care and what is research.

No known expense to subject aside from their time.

Payment for participation: *Describe all plans to pay subjects, either in cash, a gift or gift certificate. Please note that all payments must be prorated throughout the life of the study. The IRB will not approve a study where there is only a lump sum payment at the end of the study because this can be considered coercive. The amount of payment must be justified. Clarify if subjects will be reimbursed for travel or other expenses.*

☐ ***Not applicable***

Women in both conditions will receive compensation for completing 7 formal assessments and an 8th assessment of treatment acceptability and barriers/facilitators of treatment engagement at $50/assessment = $400. Women assigned to the Best Practices condition will have the potential to earn an additional $65 in incentives for completing the quit-line counseling. Women assigned to the Contingent Incentives condition will have the potential to earn the additional $65 in incentives for completing the quit-line incentives plus incentives in the form of money loaded onto a True Link or PEX Debit Card for abstaining from smoking. The incentives that participants earn will vary depending on how early in the pregnancy she enters the study and how well she abstains from smoking. However, the maximum potential amount that a woman could earn is approximately $1620 in incentives. This total is equivalent to the maximum total earnings in our prior trials adjusted for inflation ($1,200 in 2002 USD = $1,600 today).

Collaborating Sites*. When research involving human subjects will take place at collaborating sites or other performance sites when UVM/UVM Medical Center is the lead site, the principal investigator must provide in this section a list of the collaborating sites and their Federalwide Assurance numbers when applicable. (agreements may be necessary)*

☐ ***Not applicable***

As noted previously, in addition to the main trial, we will recruit an additional 60 AI/AN pregnant women at a collaborating site. That site is the Alaska Native Tribal Health Consortium (ANTHC), Anchorage Alaska. The Alaska Area IRB (AAIRB) FWA is IRB 00000636. The Site Principal Investigator for the ANTHC is Dr. Kathy Koller, Ph.D. Dr. Koller's research focuses on developing novel, theory-based behavioral interventions for tobacco cessation, including NIH-supported intervention programs to reduce tobacco disparities among AI/AN adolescents and pregnant women. The co-investigators at the ANTHC are Dr. Timothy Thomas, MD (ANTHC), Dr. Christi Patten, PhD (Mayo Clinic, Rochester, MN), and Dr. Steven Steinbubl, MD (ANTHC). University of Vermont PI Allison Kurti, PhD, is in the process of obtaining separate IRB approvals as mandated by the ANTHC to collect study data among a smaller subset of AI/AN women who are randomized separately to the Incentives versus Best Practices treatment conditions.

INFORMED CONSENT

Consent Procedures*: Describe the consent procedures to be followed, including the circumstances under which consent will be obtained, who will seek it, and the methods of documenting consent. Specify the form(s) that will be used e.g. consent (if multiple forms explain and place identifier on each form), assent form and/or HIPAA authorization (if PHI is included). These form(s) must accompany the protocol as an appendix or attachment.*

Note: Only those individuals authorized to solicit consent may sign the consent form confirming that the prospective subject was provided the necessary information and that any questions asked were answered.

With respect to the informed consent process, eligible participants will be given detailed information about the study including the following: (a) each study condition; (b) compensation for their time (e.g., completing assessments); (c) the process of randomization and the equal chance of being assigned to one of the two study conditions; (d) protection of confidentiality and the right to withdraw at any time; (e) expectations regarding the completion of formal assessments during and following their delivery (regardless of smoking status); (f) risks and benefits of study participation; (g) the need for their permission to recruit and collect a few pieces of medial record data pertaining to their infant; and (h) our procedures for dealing with any endorsement of suicidality. Participants who self-refer via

CHRMS (Medical) #17-0604 Approved: 4/3/2020

print or online advertisements will also be given information about the need to verify their pregnancy status with their healthcare provider or local WIC director prior to enrolling them in the study, and will be asked to provide an electronic signature on a medical release form allowing us to verify their current pregnancy status prior to completing the informed consent process. Contact information for the Project Director (Dr. Kurti) and contact person at the UVM IRB will be provided during the verbal consent as well as included in the paper copy of the Research Information Sheet that is mailed to subjects' homes. All information about the study will be written at an 8th grade reading level to ensure adequate comprehension. Participants will be competent adults who can provide their voluntary informed consent. The informed consent process will be completed over the phone, and we will use the attached Consent & Authorization Process Documentation form to document two processes: (a) Mom's consent to participate in a smoking cessation study, and (b) Mom's permission allowing recruitment and collection of birth outcome data pertaining to her infant. Researchers will read the attached Research Information Sheet to participants and they will be provided the opportunity to ask questions or postpone providing their verbal consent to another time if they need more time to decide whether to participate.

After answering any questions, the participant may provide verbal consent to participate and for the infant to participant. Researchers will mail a paper copy of the full consent form that is mailed to participants along with equipment to participate in the study. A recent study in JAMA demonstrated the feasibility of completing the informed consent process remotely using Smartphones (McConnell et al., 2017).

The smaller subset of AI/AN women recruited at the collaborating site will undergo identical informed consent procedures as women in the main trial. More specifically, AI/AN women recruited at the collaborating site will complete the same informed consent phone call as women recruited for the main trial. UVM investigators will read the AI/AN women the same Research Information Sheet, and obtain their verbal consent using the same procedures as main trial participants. Likewise, verbal consent will be documented using the same Consent & Authorization Process form. Although Dr. Koller's connections to WIC/obstetrical clinics will to leveraged to obtain referrals, UVM investigators will be responsible for eligibility screenings, and for moving women through the informed consent process. Aside from (a) minor differences in the way that AI/AN pregnant women initially receive information about the trial (e.g., unique Facebook ads with text and images targeted more directly at AI/AN), and (b) separate randomization conducted among the subset of AI/AN women, all other aspects of the informed consent process and procedures experienced in the study will be identical to those described for the main trial

Information Withheld From Subjects: *Will any information about the research purpose and design be withheld from potential or participating subjects? If so, explain and justify the non-disclosure and describe plans for post-study debriefing.*

X	***Not applicable***

Attach full grant application, including budget information and/or any contract or draft contract associated with this application.
