# Supplementary material for: Effect of Smartphone-Based Financial Incentives on Peripartum Smoking Among Pregnant Individuals: A Randomized Clinical Trial
Source: JAMA Netw Open. 2022 May 13;5(5):e2211889. doi: 10.1001/jamanetworkopen.2022.11889 (PMC9107025; doi:10.1001/jamanetworkopen.2022.11889)
Supplement: Supplement 2. — eFigure. Study Flowchart [file jamanetwopen-e2211889-s002.pdf]

Supplemental Online Content

Kurti AN, Nighbor TD, Tang K, et al. Effect of smartphone-based financial incentives on peripartum smoking among pregnant individuals: a randomized clinical trial. *JAMA Netw Open*. 2022;5(5):e2211889. doi:10.1001/jamanetworkopen.2022.11889

eFigure. Study Flowchart

This supplemental material has been provided by the authors to give readers additional information about their work.

© 2022 Kurti AN et al. *JAMA Network Open.*

eFigure. Study Flowchart

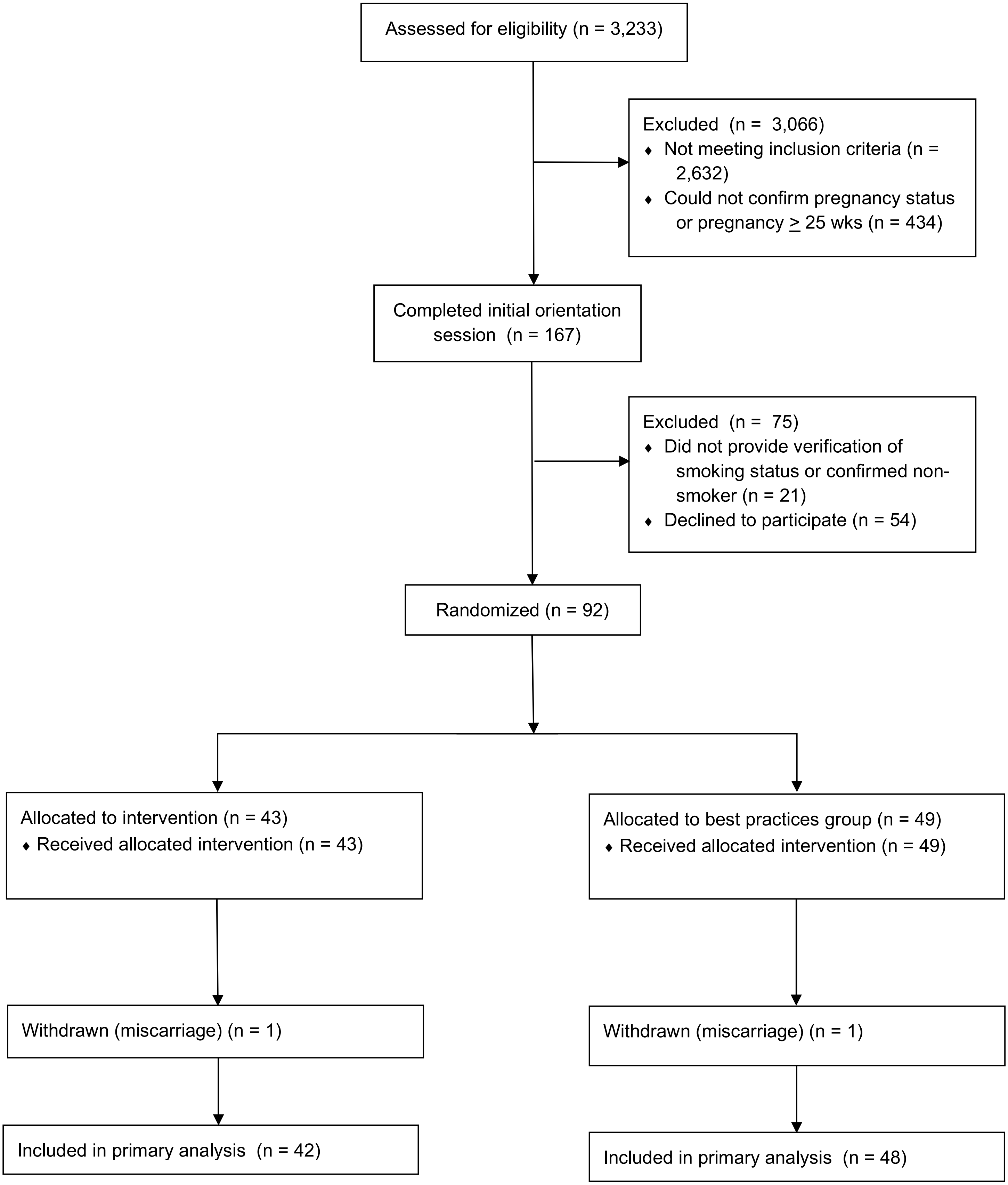

© 2022 Kurti AN et al. *JAMA Network Open.*
