# Supplementary material for: Effect of Smartphone-Based Financial Incentives on Peripartum Smoking Among Pregnant Individuals: A Randomized Clinical Trial
Source: JAMA Netw Open. 2022 May 13;5(5):e2211889. doi: 10.1001/jamanetworkopen.2022.11889 (PMC9107025; doi:10.1001/jamanetworkopen.2022.11889)
Supplement: Supplement 3. — Data Sharing Statement [file jamanetwopen-e2211889-s003.pdf]

Data Sharing Statement

Kurti. Effect of Smartphone-Based Financial Incentives on Peripartum Smoking Among Pregnant Individuals. *JAMA Netw Open*. Published May 13, 2022.
doi:10.1001/jamanetworkopen.2022.11889

Data
Data available: Yes
Data types: Deidentified participant data
How to access data: akurti@uvm.edu
When available: With publication

Supporting Documents
Document types: None

Additional Information
Who can access the data: researchers whose proposed use of the data has been approved
Types of analyses: for a specified purpose
Mechanisms of data availability: after approval of a proposal
